# Experimental population modification of the malaria vector mosquito, *Anopheles stephensi*

**DOI:** 10.1371/journal.pgen.1008440

**Published:** 2019-12-19

**Authors:** Thai Binh Pham, Celine Hien Phong, Jared B. Bennett, Kristy Hwang, Nijole Jasinskiene, Kiona Parker, Drusilla Stillinger, John M. Marshall, Rebeca Carballar-Lejarazú, Anthony A. James

**Affiliations:** 1 Department of Microbiology & Molecular Genetics, University of California, Irvine, California, United States of America; 2 Biophysics Graduate Group, University of California, Berkeley, California, United States of America; 3 Division of Epidemiology & Biostatistics, School of Public Health, University of California, Berkeley, California, United States of America; 4 Innovative Genomics Institute, Berkeley, California, United States of America; 5 Department of Molecular Biology & Biochemistry, University of California, Irvine, California, United States of America; New York University, UNITED STATES

## Abstract

Small laboratory cage trials of non-drive and gene-drive strains of the Asian malaria vector mosquito, *Anopheles stephensi*, were used to investigate release ratios and other strain properties for their impact on transgene spread during simulated population modification. We evaluated the effects of transgenes on survival, male contributions to next-generation populations, female reproductive success and the impact of accumulation of gene drive-resistant genomic target sites resulting from nonhomologous end-joining (NHEJ) mutagenesis during Cas9, guide RNA-mediated cleavage. Experiments with a non-drive, autosomally-linked malaria-resistance gene cassette showed ‘full introduction’ (100% of the insects have at least one copy of the transgene) within 8 weeks (≤ 3 generations) following weekly releases of 10:1 transgenic:wild-type males in an overlapping generation trial design. Male release ratios of 1:1 resulted in cages where mosquitoes with at least one copy of the transgene fluctuated around 50%. In comparison, two of three cages in which the malaria-resistance genes were linked to a gene-drive system in an overlapping generation, single 1:1 release reached full introduction in 6–8 generations with a third cage at ~80% within the same time. Release ratios of 0.1:1 failed to establish the transgenes. A non-overlapping generation, single-release trial of the same gene-drive strain resulted in two of three cages reaching 100% introduction within 6–12 generations following a 1:1 transgenic:wild-type male release. Two of three cages with 0.33:1 transgenic:wild-type male single releases achieved full introduction in 13–16 generations. All populations exhibiting full introduction went extinct within three generations due to a significant load on females having disruptions of both copies of the target gene, *kynurenine hydroxylase*. While repeated releases of high-ratio (10:1) non-drive constructs could achieve full introduction, results from the 1:1 release ratios across all experimental designs favor the use of gene drive, both for efficiency and anticipated cost of the control programs.

## Author summary

The experimental introduction of manipulated genes into insect species has a long history in basic genetics. Recent advances in genome editing technologies have spurred considerable effort to exploit these methodologies to provide genetic solutions to some of the worst medical and agricultural problems caused by insects. Insect population suppression and population modification approaches have been proposed to control transmission of vector-borne diseases, including malaria. We used small cage trials to explore the efficacy of non-drive and gene-drive releases to deliver anti-malarial effector genes to a vector mosquito, *Anopheles stephensi*. We show that both approaches can work to introduce genes to high percentages, but as expected, the gene-drive approaches were more efficient in that they needed only a single release with a much lower number of released insects. The gene-drive females in our studies exhibited a significant load that resulted in some cage populations going to extinction. Furthermore, the accumulation of drive-resistant target genes prevented full introduction of the transgenes in those cages that did not go extinct. While none of the strains evaluated here are proposed for open release, these laboratory cage trials reveal features that can be used to improve next-generation gene-drive strains for population modification.

## Introduction

Mosquito-borne diseases continue to be one of the greatest challenges to global health. Recent decreases in malaria morbidity and mortality have reversed, and viral diseases, including dengue and chikungunya fever and Zika, remain largely unchecked [1–3]. While efforts in vaccine development and mass drug administration continue, vector control remains the most significant and cost-effective way to protect populations from malaria epidemics [4, 5]. However, insecticide resistance is threatening current gains [6, 7] and this has fostered a number of research efforts to develop genetic strategies to control malaria transmission [8, 9].

There are two categories of genetic vector control strategies, the first of which, population suppression, comprises genetic analogs of insecticides, source reduction and other methods designed to reduce or eliminate local vector populations. The second, population modification, seeks to alter the ability of a vector mosquito to transmit pathogens. Considerable success with proofs-of-principle have been demonstrated for both approaches [9, 10]. Each has a long theoretical, and in some cases, practical history in vector control, but the adoption of molecular genetic technologies, including DNA cloning and transgenesis, have brought many of the more speculative approaches closer to applied end-products [11–14]. Indeed, a population suppression technology has been tested in field trials [15, 16].

Unlike vaccines, drugs and insecticides, the pathways from laboratory discovery through development and eventually delivery of a genetically-engineered vector control product have yet to be fully defined and tested. Efforts to identify and adopt standards for product efficacy and safety resulted in several documents developed by the proponents of the technologies [17–21]. These were sufficient to allow some countries to issue permits for the releases in open field trials of self-limiting suppression strains of the yellow fever mosquito, *Aedes aegypti*, and the African malaria vector, *Anopheles gambiae* [15, 16, 22]. However, laboratory demonstrations of powerful genetic systems for altering vector genomes have attracted attention from independent agencies such as the National Academies of Sciences, Engineering and Medicine (NASEM, USA), World Health Organization (WHO) and others, and they have endorsed a phased approach to testing these new products [23–25]. Included in these are recommendations for laboratory cage trials with requirements that need to be met before moving products to the next phase.

Population modification (also known as replacement or alteration) requires the introduction of genes that confer resistance to one or more target pathogens into a mosquito species [8, 10]. Population modification could be achieved by ‘inundative release’ of non-drive, pathogen-resistant strains in which serial applications of large numbers of insects carrying genes is expected eventually to result in every individual in the target population carrying the beneficial traits [11]. The logistics of rearing, releasing and monitoring large numbers of genetically-engineered mosquitoes has been demonstrated already for a population suppression strain [15, 16]. However, the speed at which the genes are introduced is expected to be higher and the cost of application lower if a genetic mechanism, a so-called ‘gene-drive’, was used [8, 10]. We used a series of small cage trials of both non-drive and gene-drive strains of the Asian malaria vector mosquito, *Anopheles stephensi*, to probe some of the parameters of release ratios and other factors for their impact on transgene introduction. Although none of the strains tested herein will ever be released, lessons learned from these small cage experiments can inform the design of both next-generation gene drive systems and the phase testing needed for further development of the technology.

## Methods

### Mosquito strains

A colony of *Anopheles stephensi* (Indian Strain, gift of M. Jacobs-Lorena, Johns Hopkins University) maintained at the University of California, Irvine (UCI) insectary for >15 years is the source of all insects used in the experiments. Transgenic and wild-type (non-transgenic) mosquitoes were maintained at 27°C with 77% humidity and a 12-hour day/night, 30 min dusk/dawn lighting cycle. Larvae were fed a diet of powdered fish food (Tetramin, Melle, Germany) mixed with yeast. Adults were provided water and a 10% sucrose solution *ad libitum*. Routine bloodmeals for females consisted of calf’s blood (Colorado Serum Company, CO) provided from a feeding apparatus (Hemotek, Inc., Blackburn, UK). Anesthetized mice were used to provide bloodmeals in 0.216 m^3^ (60 X 60 X 60 cm) cage formats.

The AP26 transgenic line was created by linking dual anti-malarial single-chain antibody (scFv) genes in a ‘tail-to-tail’ orientation flanked by *gypsy* insulator sequences [26], and cloning them adjacent to *loxP* sites flanking a 3xP3-DsRed marker gene (S1 Fig). The dual anti-parasite effector genes are based on scFvs, m1C3 and m2A10, derived from monoclonal antibodies that target the human malaria parasite, *Plasmodium falciparum* ookinete protein Chitinase 1 and the circumsporozoite protein (CSP), respectively [27–29]. These genes were cloned into a plasmid containing an *attB* site for *φC31* phage recombinase-mediated site-specific integration. The resulting plasmid was injected into a ‘docking site’ line, attp26 10.1, created previously in our laboratory by *piggyBac* transposon-mediated insertion of a transgene construct, pBac[3xP3-ECFPfa]*attP* [30], into the genome of *An*. *stephensi*. Southern blot analysis based on previously-published protocols, restriction endonuclease-digested genomic DNA, and a DNA probe complementary to the enhanced cyan fluorescent protein (ECFP) open reading frame were used to confirm transgene copy number [27, 31, 32]. Inverse PCR techniques [27] were used to identify the chromosomal location of the docking site. Microinjection and screening procedures are described in [33] and [34]. The resulting line, AP26, was made homozygous and assayed by reverse transcriptase-based gene amplification (RT-PCR) for expression of the dual scFvs.

The AsMCRkh2 10.1 line (abbreviated hereafter as AsMCRkh2) contains the dual scFvs described above linked to an autonomous gene drive construct based on Cas9 biology [13] and is maintained by continuous outcrossing of transgenic males to wild-type females. AsMCRkh2 is marked with DsRed and has a guide RNA (kh2) that targets the wild-type copy of the *kynurenine hydroxylase-white* (*kh*) gene (also known as *kynurenine monooxygenase*) located autosomally on chromosome 3L [13, 35]. Disruptions of the *kh* gene cause a recessive white-eye phenotype (*kh*^*w*^)in *An*. *stephensi* [13].

### Non-drive (ND) release trials

Sixty mixed-sex, second-instar (L2), wild-type larvae were placed over three successive weeks into each of nine 0.216 m^3^ cages to create age-structured populations (S2 Fig). Adult females were provided at week 3 with anesthetized mice as a bloodmeal source and an oviposition container. Following that, females were bloodfed once weekly and provided oviposition containers. Eggs from each cage were hatched weekly and 60 L2 larvae were selected at random and returned to their respective cages to offset mortality (weeks 4–8).

Cages were assigned randomly in 3 triplicate sets at week 9 as ‘ND-Control-A, B and C’, ‘ND-1:1-A, B and C’ and ‘ND-10:1-A, B and C’. ‘ND’ refers to ‘non-drive’, 1:1 and 10:1 refer to transgenic to wild-type male release ratios, and ‘A’, ‘B’ and ‘C’ refer to the individual cage replicates. As before, females were provided mice for a bloodmeal and an oviposition container. Eggs collected from each cage were hatched and mosquitoes were allowed to develop into pupae. Sixty wild-type (30 male and 30 female) pupae were added weekly to each of the nine cages to replenish the populations following weekly die-offs of adults in the cages. All ND 1:1 (A, B and C) cages had an additional 30 transgenic AP26 male pupae added at the same time to create a 1:1 AP26:wild-type male release ratio while cages ND-10:1-A, B and C had an additional 100 AP26 male pupae added on three consecutive days (for a total of 300 AP26 male pupae) to create an overall weekly 10:1 AP26:wild-type male release ratio. A total of 300 larvae resulting from the output of each cage were selected at random starting at week 13 and screened for the DsRed marker gene (DsRed-positive [DsRed^+^]), which indicates the presence of the AP26 transgene. These animals then were reared to adults to determine their sex. This procedure, including the addition of AP26 males, was repeated through week 22.

### Overlapping generation gene-drive (OD) release trials

Two sets of triplicate 0.216 m^3^ cage populations were set up for overlapping generation gene-drive (OD) experiments with Cages OD-1:1 (A, B and C) founded by adding to each 120 wild-type male, 120 AsMCRkh2 male and 120 wild-type female pupae for a 1:1 male release ratio (S3 Fig). Cages OD-0.1:1 (A, B and C) each had 120 wild-type male, 12 AsMCRkh2 male and 120 wild-type female pupae added for a 0.1:1 male release ratio. Females in each cage were provided mice for bloodmeals and an oviposition container per generation (~3 weeks). Eggs were hatched and 240 first-instar (L1) larvae from each cage chosen at random and returned to their respective cages. No additional AsMCRkh2 males were added during any of the subsequent generations. A total of 300 larvae from each cage also were selected at random and screened for phenotypes at the larval (DsRed-positive [DsRed^+^] or DsRed-negative [DsRed^-^]), pupal (wild-type or white-eyed phenotypes [*kh*^*w*^]) and adult stages (for sex). This protocol was followed for seven generations, each lasting ~3 weeks and delimited by the bloodmeal.

### Non-overlapping generation gene-drive (NOD) release trials

These trials consisted of non-overlapping generations (NOD) of gene-drive mosquitoes where progeny were not returned to their cages as they were in the overlapping (OD) release trials, but rather added to a separate cage from their parents (S4 Fig). Three sets of triplicate small cage (0.005 m^3^) populations were set up with 100 wild-type female pupae each and the following numbers and ratios of transgenic AsMCRkh2:wild-type male pupae: Cages NOD-1:1 (A, B and C) had 50:50 AsMCRkh2:wild-type, Cages NOD-0.33:1 (A, B and C) had 25:75 AsMCRkh2:wild-type, and Cages NOD-0.1:1 (A, B and C) had 9:90 AsMCRkh2:wild-type. No additional AsMCRkh2 males were added at any subsequent generation. Mosquitoes were reared to the adult stage and females were provided a blood meal 5 d postemergence using the Hemotek feeding apparatus. Dead adults were removed and oviposition containers were provided. After 3 d, the oviposition containers were removed, and the remaining adults were counted by sex and frozen at -80°C.

Larvae were hatched from the oviposition containers and 200 L1 selected randomly from the NOD-1:1 and NOD-0.33:1 cages were used to populate new cages for the next generation. All larvae from generations 1–12 of the NOD-0.1:1 cages were scored for the DsRed eye-color marker for transgene frequency, and 200 larvae reflecting the existing transgene gene frequency were used to populate new cages. NOD-0.1:1 cages were maintained identically to NOD-1:1 and NOD-0.33:1 cages after generation 12.

Following the removal of the 200 larvae for the establishment of the next generation cages, all remaining larvae were screened and scored for the DsRed phenotype. Approximately 500 of these larvae were selected randomly, reared to pupae, and scored for the target gene eye-color phenotype. The following phenotypes were recorded: wild-type (DsRed^-^/*kh*^*+*^), DsRed black eye (DsRed^+^/*kh*^*+*^), DsRed white-eye (DsRed^+^/*kh*^*w*^), white-eye only (DsRed^-^/*kh^w^*) and two types of mosaic-eyes that were scored as the same (DsRed^+^/*kh*^*mosaic*^). Pupae were reared to adults and counted and scored by sex. Two ‘exceptional’ phenotypes (white-eye only [DsRed^-^/*kh*^*w-*^] and later generation ‘wild-type’ [DsRed^-^/*kh*^*+*^] mosquitoes) were to be molecularly analyzed, and were outcrossed with wild-type mosquitoes of the opposite sex and parental DNA preserved for subsequent gene amplification (PCR) analyses.

### Gene amplification analysis

Genomic DNA was isolated from adult mosquitoes with exceptional phenotypes using the SYBR Green Extract-N-Amp Tissue PCR Kit (Sigma). Two oligonucleotide primers (5’GTCCACTAACGAAAGAGGTCAAGAGC3’ and 5’CGATCGTTTAGTGACGAGATCACGC3’) [13] designed to amplify a DNA fragment of 683 base pairs (bp) in length were used to characterize the *kh* locus for mutations at the Cas9 target site. The nucleotide sequences of the amplified fragments were obtained commercially and aligned with the wild-type sequence.

### White-eye female phenotypes

Females with white eyes can be homozygous for the AsMCRkh2 drive construct, homozygous for nonfunctional *kh* alleles resulting from non-homologous end joining (NHEJ), heteroallelic for NHEJ alleles, or heterozygous for the drive construct and a NHEJ allele. Females with these genotypes were generated by intercrossing ten replicates each of AsMCRkh2/*kh*^*+*^ (10 males and 10 females) or AsMCRkh2/*kh*^*w*^ (10 males and 10 females). Following a blood meal, segregating heterozygous AsMCRkh2/*kh*^*+*^ or homozygous *kh*^*w*^ females were compared with wild-types from the same crosses for survival, number of females laying eggs, average number of eggs per female laid and survival of eggs to larval stage. Females were given a blood meal using the Hemotek feeder on two consecutive days at 5 d post adult emergence and survival recorded after 3 d. The fertility of each female was recorded after 5 d. All progeny were hatched and survival recorded at day 6.

### Modeling cage population dynamics

Empirical data from the non-overlapping gene drive trials were used to parameterize a model of CRISPR-based homing gene drive including resistant allele formation, and a stochastic implementation of the fitted model was used to compare the frequencies of observed population extinctions to model-predicted ones. Model fitting was carried out for all nine cages in non-overlapping gene drive experiments using Markov chain Monte Carlo (MCMC) methods in which estimated parameters included allele-specific fitness costs and the consequences of maternal deposition of Cas9.

We considered discrete generations, random mixing, and Mendelian inheritance rules at the gene drive locus, with the exception that, for adults heterozygous for the homing allele (denoted by “H”) and wild-type allele (denoted by “W”), a proportion, *c*, of the W alleles are cleaved, while a proportion, 1 − *c*, remain as W alleles. Of those that are cleaved, a proportion, *p*_*HDR*_, are subject to accurate homology-directed repair (HDR) and become H alleles, while a proportion, 1 − *p*_*HDR*_, become resistant alleles. Of those that become resistant alleles, a proportion, *p*_*RES*_, become in-frame, cost-free resistant alleles (denoted by “R”), while the remainder, 1 − *p*_*RES*_, become out-of-frame or otherwise costly resistant “broken” alleles (denoted by “B”). The value of *p*_*HDR*_ is allowed to vary depending on whether the HW individual is female or male, and values for female and male-specific parameters were estimated based on first-generation, post-release, progeny that provided direct information on them.

The effects of maternal deposition of Cas9 were accommodated after computing the gene drive-modified Mendelian inheritance rules. If offspring having a W allele had a mother having the H allele, then this would lead to Cas9 being deposited in the embryo by the mother, possibly resulting in cleavage of the W allele. We considered cleavage to occur in a proportion, *p*_*MC*_, of these embryos, with a proportion, *p*_*MR*_, of the cleaved W alleles become R alleles, and the remainder, 1 − *p*_*MR*_, becoming B alleles.

These considerations allow us to calculate expected genotype frequencies in the next generation, and to explore the fitness and maternal deposition parameters that maximize the likelihood of the experimental data. Estimated parameters include multiplicative fitness costs associated with having one copy of the H, R and B alleles, and *p*_*RES*_, *p*_*MC*_ and *p*_*MR*_, as defined earlier. Female genotypes HH, HB and BB were assumed to be infertile based on experimental data. A stochastic version of the fitted model was implemented using a discrete generation version of the Mosquito Gene Drive Explorer model (MGDrivE [36]) with an adult population size of 600. The complete modeling framework is described in the Supporting Information (S1 Text).

### Animal ethics statement

This study was carried out in strict accordance with the recommendations in the Guide for the Care and Use of Laboratory Animals of the National Institutes of Health. Protocols were approved by the Institutional Animal Care and Use Committee of the University of California (Animal Welfare Assurance Numbers A3416.01).

## Results

### Non-drive introduction of anti-malaria genes into caged wild mosquito populations

Several *An*. *stephensi* transgenic lines were generated in which *piggyBac* transposon-mediated transformation was used to insert *attP* DNA sequences into the mosquito genome to serve as ‘docking sites’ for *φC31* phage recombinase-mediated site-specific integration [31]. Once such line, attp26 10.1, was shown by Southern blot analysis and inverse PCR to have a single insertion into the autosome, 2L, at a location that did not appear to encode any gene (including putative promoter and transcribed regions) or other recognizable transcribed DNA (S1 Fig). Simple crossing created a strain that was homozygous in both males and females for the docking-site transgene. We then used *φC31* recombinase to integrate the m1C3 and m2A10 scFv transgenes [27–29, 32] into this site to yield a line, AP26, from which it was possible to generate males containing two copies of the transgenes for the non-drive experiments. Transcription of the scFvs was demonstrated with RT-PCR but the line was not tested in parasite challenge assays in these experiments.

**Fig 1:**
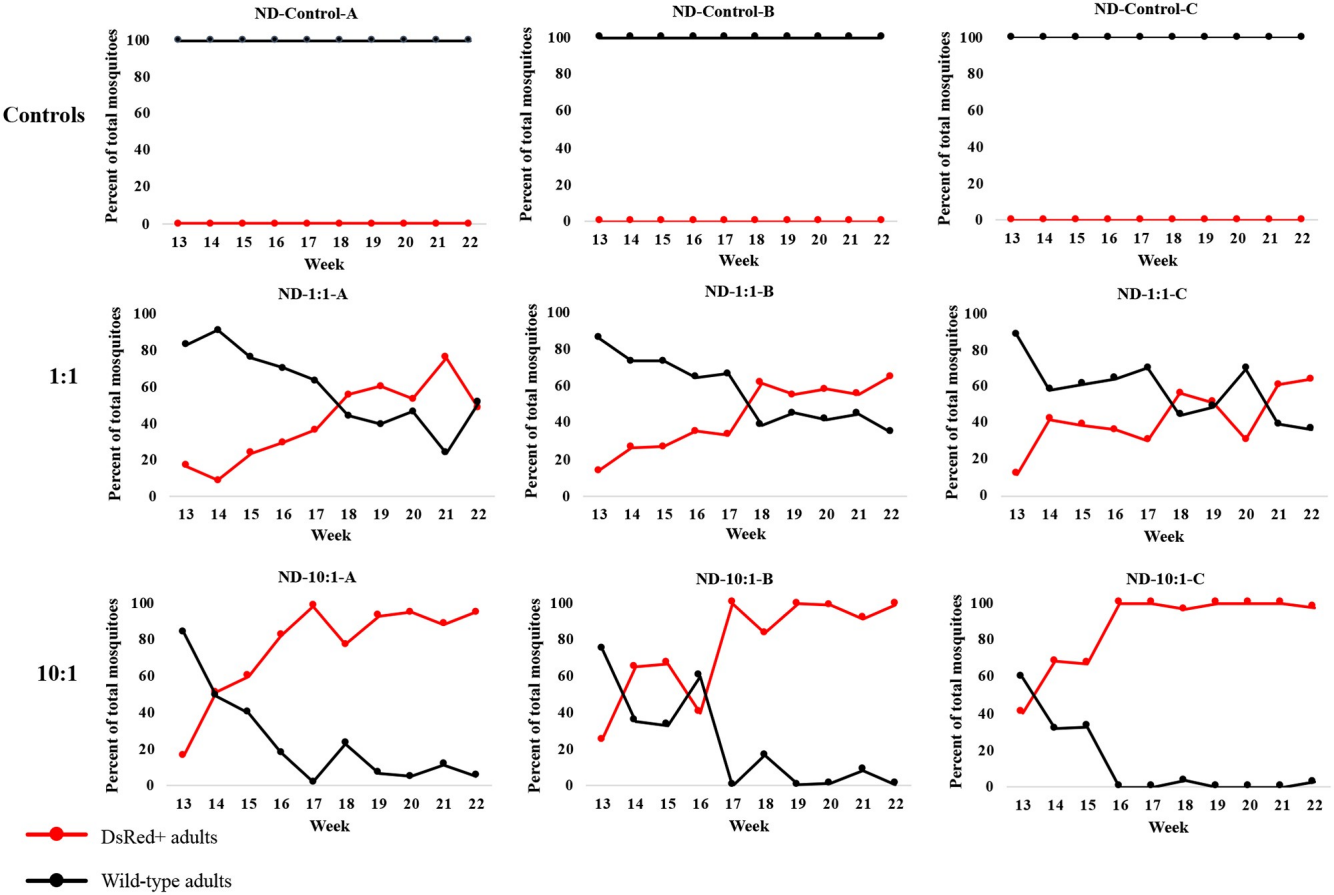
Adult phenotypes in line AP26 non-drive (ND) release cage trials. Three sets of triplicate cages (ND-Control-A, -B and -C; ND-1:1-A, -B and -C; ND-10:1-A, -B and -C) were set up with stable populations of different ages of wild-type (WT) *Anopheles stephensi* mosquitoes. AP26 transgenic males were added in the ratios indicated (Controls, none; 1:1 and 10:1) starting at week 13 and continuing weekly throughout the experiments. A total of 300 randomly-selected larvae and the resulting adults from the progeny of each cage were screened weekly as DsRed-positive (DsRed^+^, carrying the AP26 transgene; red circles and lines) or wild-type (no transgene; black circles and lines). The X-axis is the week number following the start of the cages and the Y-axis is the percent of total adults with the DsRed^+^ or wild-type phenotype.

Three sets of triplicate 0.216 m^3^ cages with stable, age-structured *An*. *stephensi* populations were set up to investigate the introduction of the cassette comprising the dominant DsRed marker gene and the DNA encoding the anti-malaria effector molecules into a controlled wild-type population (S2 Fig). Initial age-structured populations were maintained in the cages for eight weeks prior to the addition of transgenic AP26 male pupae. As expected, control cages showed no AP26 DsRed^+^ mosquitoes as none were ever added (Fig 1, S1–S3 Tables). Cages ND-1:1 (A, B and C) with 1:1 release ratio show the percent of AP26 DsRed^+^ transgenic mosquitoes generally increasing with a maximum of 76% (152/200) at week 21 in Cage ND-1:1-A (Fig 1, S4–S6 Tables). However, Cage ND-1:1-A fell to 48% (131/271) in the final week. We expect that since AP26 males were being introduced into cage populations with pre-existing wild-type males, it would take at least one generation after introduction before virgin wild-type females would be available for mating with transgenic males. This appears to have occurred by week 13, the time we started monitoring the cages and five weeks (≤ 2 generations) after introduction of transgenic males, after which there is a gradual increase in DsRed^+^ mosquitoes varying from 48–65%.

Two of three cages with 10:1 AP26:wild-type male releases (ND-10:1-B and ND10:1-C) achieved 95–99% DsRed^+^ during weeks 16–17, eight weeks (~ 3 generations) after the first introductions of transgenic mosquitoes (Fig 1, S8 and S9 Tables). Wild-type mosquitoes appeared in later generations in both cages with variable frequencies (for example, ND-10:1-B had 16% [48/299] and 0.003% [1/300] at weeks 18 and 19, respectively). Cage ND-10:1-A also achieved a high level of introduction, ~95% (219/231) and showed late generation wild-type mosquitoes. (Fig 1 and S7 Table).

Not all larvae in these experiments survived to be counted as adults (Table 1, S1–S9 Tables). However, there was no consistent bias for preferential survival of transgenic or wild-type animals. No significant differences in larval-to-adult survival percentages were observed between DsRed^+^ and wild-type mosquitoes across all cages (Table 1, t-test, p = 0.64, n = 15). The same also was observed in each single cage across all generations (S10–S12 and S14–S16 Tables).

**Table 1:** Larval to adult survival in non-drive cage trials^1^.

	DsRed^+^		DsRed^-^ (wild-type)	
Cage (release ratio^2^)	Larvae	Adults (%)^3^	Larvae	Adults (%)^3^
**ND-Control-A (none)**	-	-	3000	2681 (89)
**ND-Control-B (none)**	-	-	3000	2746 (92)
**ND-Control-C (none)**	-	-	2553	2274 (89)
**ND-1:1-A (1:1)**	1264	1013 (82)	1716	1479 (86)
**ND-1:1-B (1:1)**	1321	1072 (81)	1667	1434 (86)
**ND-1:1-C (1:1)**	1261	1058 (84)	1742	1448 (83)
**ND-10:1-A (10:1)**	2296	1770 (77)	735	598 (81)
**ND-10:1-B (10:1)**	2095	1731 (83)	587	519 (88)
**ND-10:1-C (10:1)**	2423	2124 (88)	462	295 (64)

^1^Data derived from S1–S9 Tables and are cumulative for all generations.

^2^Transgenic:wild-type males

^3^No significant differences in larval-to-adult survival percentages between DsRed^+^ and DsRed^-^mosquitoes (unpaired t-test, p = 0.64, n = 15)

### Gene drive-mediated introduction of anti-malaria genes into wild mosquito populations

#### Overlapping generation gene-drive (OD) release trials

Previous work with the AsMCRkh2 strain, which contains an autonomous gene-drive system with Cas9 driven by the *vasa* promoter, a U6 promoter-driven gRNA that targets the *kynurenine hydroxylase* (*kh*) locus on chromosome 3L linked to the dual antimalarial single chain antibodies, m1C3 and 2A10, had shown a high efficiency of drive as the progeny of outcrosses between putative heterozygous transgenic males and wild-type females were ~99.5% DsRed^+^, indicating that most contained at least one copy of the drive allele following near complete drive in the male parental germline [13]. A series of overlapping generation 0.216 m^3^ cage experiments were carried out to get a preliminary assessment of the rate of introduction of the drive construct into wild-type cage populations (S3 Fig). Unlike the previous non-drive release experiments, these overlapping gene-drive cage trials had only a single introduction of AsMCRkh2 male pupae along with 240 WT (120 male and 120 female) pupae at the initial establishment of the cage population. The 2:1 male:female ratio was anticipated to mimic what might be achievable in an actual release trial. Furthermore, cages were not set up with age-structure and the data collection was done at every generation (~ 3 weeks) instead of weekly as in the non-drive trial.

Cages OD-1:1 (A, B and C) were set up with a 1:1 AsMCRkh2 transgenic:wild-type male release ratio. Screening of first-generation post-release larvae showed 36% (108/300), 51% (154/300) and 48% (145/300) DsRed^+^ animals in cages OD-1:1-A, B and C, respectively (S5 Fig, S10–S12 Tables). We expect ~50% DsRed^+^ larvae per cage based on the previously observed drive rate of 99.5% [13] if the transgenic males could contribute equally with wild-type males to the next generation following their emergence as adults, and if the majority of females in the cages were equally receptive to mating either type (they had not mated previously because they were introduced as pupae). Cages OD-1:1-B and C are consistent with equal contributions (*Χ*^2^ = 0.106 [p = 0.744049] and 0.166 [p = 0.683691], respectively, not significant at p < 0.01). Cage OD-1:1-A showed a significantly lower contribution of the transgenic males to the first generation (*Χ*^2^ = 11.76 [p = 0.000605]).

**Fig 2:**
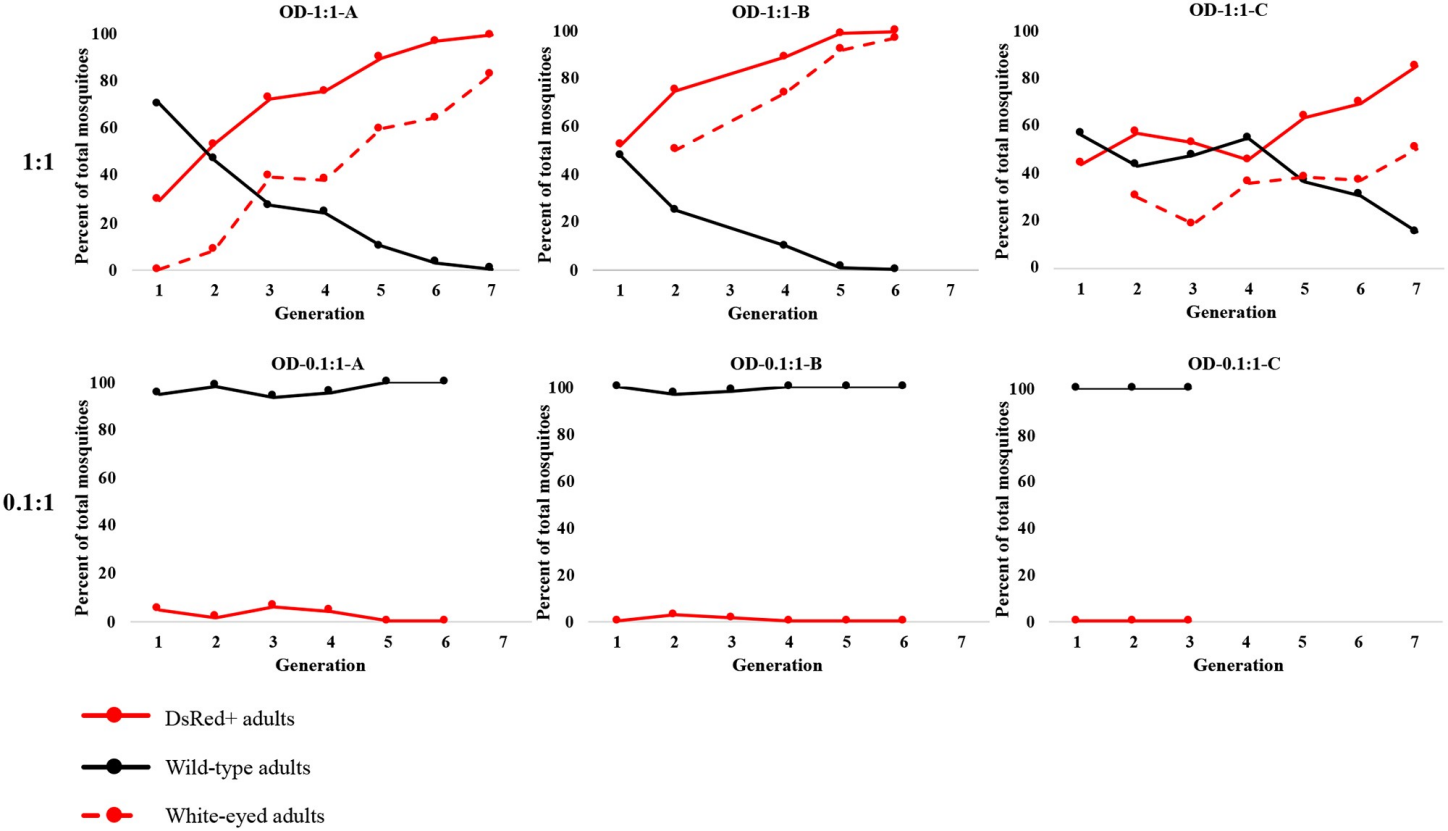
Adult phenotypes in an AsMCRkh2 overlapping gene drive (OD) cage trial. Two sets of triplicate cages (OD-1:1-A, -B and -C; OD-0.1:1-A, -B and -C) were set up with wild-type *Anopheles stephensi* pupae and the indicated ratios (1:1; 0.1:1) of AsMCRkh2 to wild-type male pupae. Larvae and resulting adults were screened in the first generation as DsRed-positive (DsRed^+^, carrying the AsMCRkh2 gene drive cassette; red circles and lines) or wild-type (no cassette; black circles and lines). The X-axis is the generation number following the start of the cages and the Y-axis is the percentage of total adults with the DsRed^+^ or wild-type phenotype. The percentages of white eye and mosaic eye mosquitoes among the total also is shown (dotted and dashed red line).

A rapid rate of increase in DsRed^+^ mosquitoes was seen in the second generation in Cages OD-1:1-A and B, so that most mosquitoes had at least one copy of the drive construct by generation 6–7 (99.7% [260/261] and 100% [290/290], respectively) (Fig 2, S10–S11 Tables). (Recall that 100%, ‘full introduction’, is defined as all insects having at least one copy of the transgenes). Cage OD-1:1-C took four generations to start to show an increase of DsRed^+^, likely due to stochastic effects (S1 Text), but eventually also exhibited a high percentage (85% [231/272]) of transgene introduction by generation 7 (Fig 2, S12 Table). Larval-to-adult survival in both release ratios cumulative over the full course of the trials were variable among cages, but no consistent trends were evident as we saw no significant differences in survival percentages between DsRed^+^ and wild-type mosquitoes across all cages and generations (Table 2, t-test, p = 0.6, n = 11). The same also was observed in each single cage across all generation (S10–S12, S14–S16 Tables).

**Table 2:** Larval to adult survival in overlapping gene drive cage trials^1^.

	DsRed^+^		DsRed^-^ (wild-type)	
Cage	Larvae	Adults (%)^2^	Larvae	Adults (%)^2^
**OD-1:1-A**	1570	1324 (84)	537	475 (88)
**OD-1:1-B**	1536	1107 (72)	265	220 (83)
**OD-1:1-C**	1274	1100 (86)	832	726 (87)
**OD-0.1:1-A**	48	42 (88)	1752	1500 (86)
**OD-0.1:1-B**	11	11 (100)	1785	1642 (92)
**OD-0.1:1-C**	0	0	900	529 (59)

^1^Data derived from S10–S12 and S14–S16 Tables and are cumulative for all generations.

^2^No significant difference in larval-to-adult survival percentages between DsRed^+^ and DsRed^-^mosquitoes (unpaired t-test, p = 0.6, n = 11)

The appearance of mosaic- and white-eye phenotypes was monitored in DsRed^+^ adults in all cages as the experiments progressed (Fig 2, S5 Fig, S13 Table). Mosaic eye phenotypes comprise those with white- or light-colored ommatidia often with an adjacent patch of cells with near wild-type coloration (S5 Fig, Fig 2 in [13]). This phenotype is observed in the progeny of females carrying the active AsMCRkh2 gene-drive element mated with wild-type males and is proposed to result from early activity in females of the Cas9-gRNA complex on the incoming male chromosomes thereby creating somatic mosaicism [13]. The appearance of the white-eye phenotype in the second generation after introduction in the 1:1 releases is consistent with the first opportunity of the AsMCRkh2 transgene becoming homozygous or a heteroallelic combination of the transgene with a *kh*^*w*^ NHEJ allele (Fig 2, S5 Fig, S13 Table). All OD-1:1 cages showed an increase in white-eye phenotypes that followed as expected the increase in DsRed^+^ mosquitoes with Cages OD-1:1-A and OD-1:1-B nearing full introduction by generations 7 and 6, respectively. Cage OD-1:1-C had a slower rate of increase and had not reached full introduction prior to the termination of the experiment. While we cannot infer anything quantitatively from the total numbers, we can conclude that the first appearance of mosaic phenotypes (along with the white-eye phenotypes) in all OD-1:1 cages in generation 2 signals the contribution from the previous generation of females carrying the gene-drive construct that they must have inherited from their fathers (S13 Table).

Cages OD-0.1:1 (A, B and C) with a 0.1:1 AsMCRkh2 transgenic-to-wild-type male release ratios showed few changes in the frequency of DsRed^+^ mosquitoes and a gene-drive sweep was never established (Fig 2, S14–S16 Tables). The small numbers of mosquitoes with white- and mosaic-eye phenotypes in the 0.1:1 release replicates are expected based on the DsRed scoring (S13 Table). The white-eyed animals (four males, six females) seen in Cage OD-0.1:1-A in the fourth generation result from this cage showing some persistence of the gene drive within the population (S14 Table).

We confirmed the observation from previous experiments [13] that ablations of both copies of the *kh* gene resulting from homozygous or heteroallelic combinations of gene-drive construct insertions or NHEJ alleles producing a white-eye phenotype, impose a large and significant fitness cost on the females in our strain. Approximately 80% of *kh*^*w*^ *An*. *stephensi* females died two days after being given a blood meal, and the surviving females had significantly reduced fecundity (Table 3).

**Table 3:** Impact of ablations of the *kynurenine hydroxylase* gene on females *Anopheles stephensi* following a blood meal^1^.

	Wild-type	AsMCRkh2^+^/*kh*^*+*^	*kh*^*w*^/*kh*^*w*^	*p value*^2^
**Number of females surviving after blood feeding**	86/90	95/100	15/80	p <0.0001^3^ (N = 27, F = 120, df = 2,24)
	96%	95%	19%	
**Number of female laying eggs**	66/90	73/100	3/80	p<0.0001^3^ (N = 27, F = 163, df = 2,24)
	73%	73%	4%	
**Average number of eggs per laying female**	90	81	56.7	p = 0.015^3^ (N = 142, F = 4, df = 2,139)
**Surviving larvae**	>4000	>4000	30	

^1^8–10 replicates of each condition were tested. Each replicate contained 10 females and 10 males

^2^One-way Anova.

^3^Post-Hoc analysis with Tukey test reveals that *kh*^*w*^*/kh*^*w*^ females had significantly lower fertility and fecundity as well as survival rate post-bloodmeal compared to wild-type and AsMCRkh2^+^/*kh*^+^ females.

#### Non-overlapping generations gene-drive (NOD) release trials

The significant load seen in females led us to consider that cage populations could go extinct if all target sites were mutated to non-functional alleles. Therefore, a third series of experiments was set to test this and other drive features. AsMCRkh2 males were introduced in these experiments to wild-type *An*. *stephensi* populations in a discrete, non-overlapping experimental design. Each 0.005 m^3^ cage was seeded with 100 wild-type *An*. *stephensi* female and 99–100 total AsMCRkh2 and wild-type male pupae at different release ratios. Cages were set up with 1:1, 0.33:1 and 0.1:1 ratios of AsMCRkh2 males to wild-type males, respectively (S4 Fig). Each release ratio was conducted in triplicate, i.e. ‘NOD-1:1-A, B and C’, ‘NOD-0.33:1-A, B and C’, and ‘NOD-0.1:1-A, B and C’. A total of 644,501 mosquitoes were scored over the course of the one-year, 20-generation experiment.

**Fig 3:**
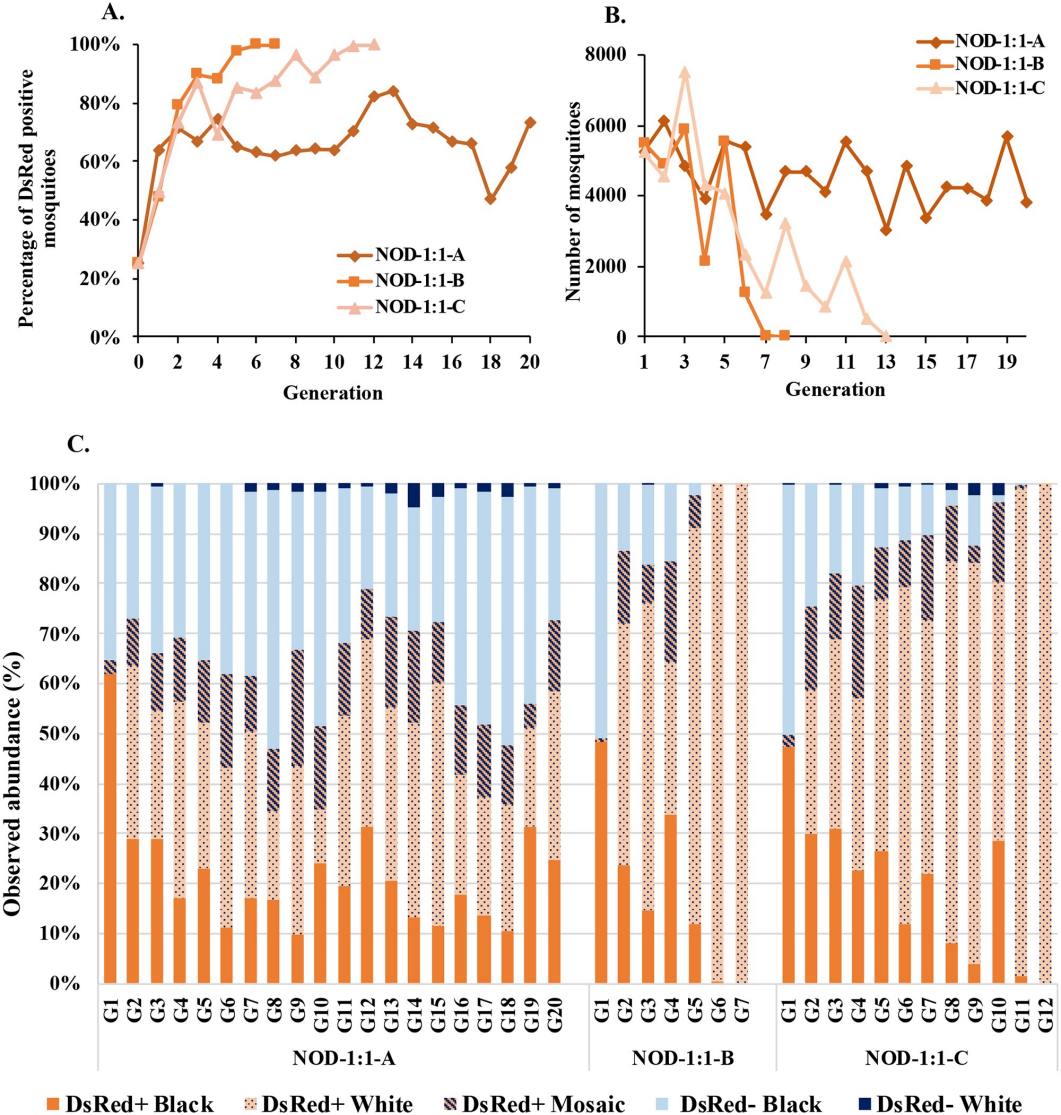
Non-overlapping gene drive (NOD) cage trial with initial releases of 1:1 transgenic AsMCRkh2 to wild-type males. Three cages (NOD-1:1A, NOD-1:1B and NOD-1:1) were seeded initially with 100 wild-type female, 50 wild-type male and 50 AsMCRkh2 male pupae. The resulting next generation larvae, pupae and adults were scored as DsRed-positive (DsRed^+^, carrying the AsMCRkh2 gene drive cassette) or wild-type. (A) Percentages of DsRed-positive (DsRed^+^) adult mosquitoes (Y-axis) in the total population in each cage scored at each generation (X-axis). (B) Total population size (Y-axis) in each replica cage at each generation (X-axis). (C) Abundance in percentages (Y-axis) of eye color and DsRed phenotypes of ~500 randomly-selected pupae per generation in each cage.

The results of the 1:1 release ratio cages were similar qualitatively through generations 6–8 to those of the previous 1:1 overlapping gene-drive trials (Fig 3, S17 and S18 Tables). Larvae scored in the first-generation following release show percentages of drive-positive animals (DsRed^+^) of 63% (NOD-1:1-A, 3227/5045), 48% (NOD-1:1-B, 2530/5291) and 49% (NOD-1:1-C, 2486/5017). Similar to the overlapping gene-drive experiments, this is consistent with equal contributions of both transgenic and wild-type males to the first generation in Cages NOD-1:1-B and NOD-1:1-C (*Χ*^2^ = 5.08 [p = 0.024] and 0.21 [p = 0.646], respectively), which should result in ~50% DsRed^+^ progeny from 99.5% drive in males at the first mating. Cage NOD-1:1-A had a significantly higher contribution of transgenic males to the first-generation progeny (*Χ*^2^ = 177.94 [p = < 0.00001]).

Cages NOD-1:1-B and NOD-1:1-C reached full introduction by generation 6 and 12, respectively, as evidenced by the DsRed^+^/*kh*^*w*^ phenotypes. The percentage of DsRed^+^ mosquitoes in Cage NOD-1:1-A remained at ~62% from generations 5–10, but then began to increase at generation 11, reaching 84% at its highest in generation 13, regressed to 47% at generation 18 and went up to 73% by the end of the experiment.

Overall population sizes fluctuated in all three cages but dropped sharply in cages NOD-1:1-B and NOD-1:1-C concomitant with the increase in frequency of ablated *kh* genes (Fig 3). This was expected given the observed severe load on *white-eyed* females following a blood meal. Both cages went to extinction as the previous generations achieved homozygosity for *kh* ablations. Cage NOD-1:1-A populations stabilized at generation 7 and stayed so until the end of the experiment.

**Fig 4:**
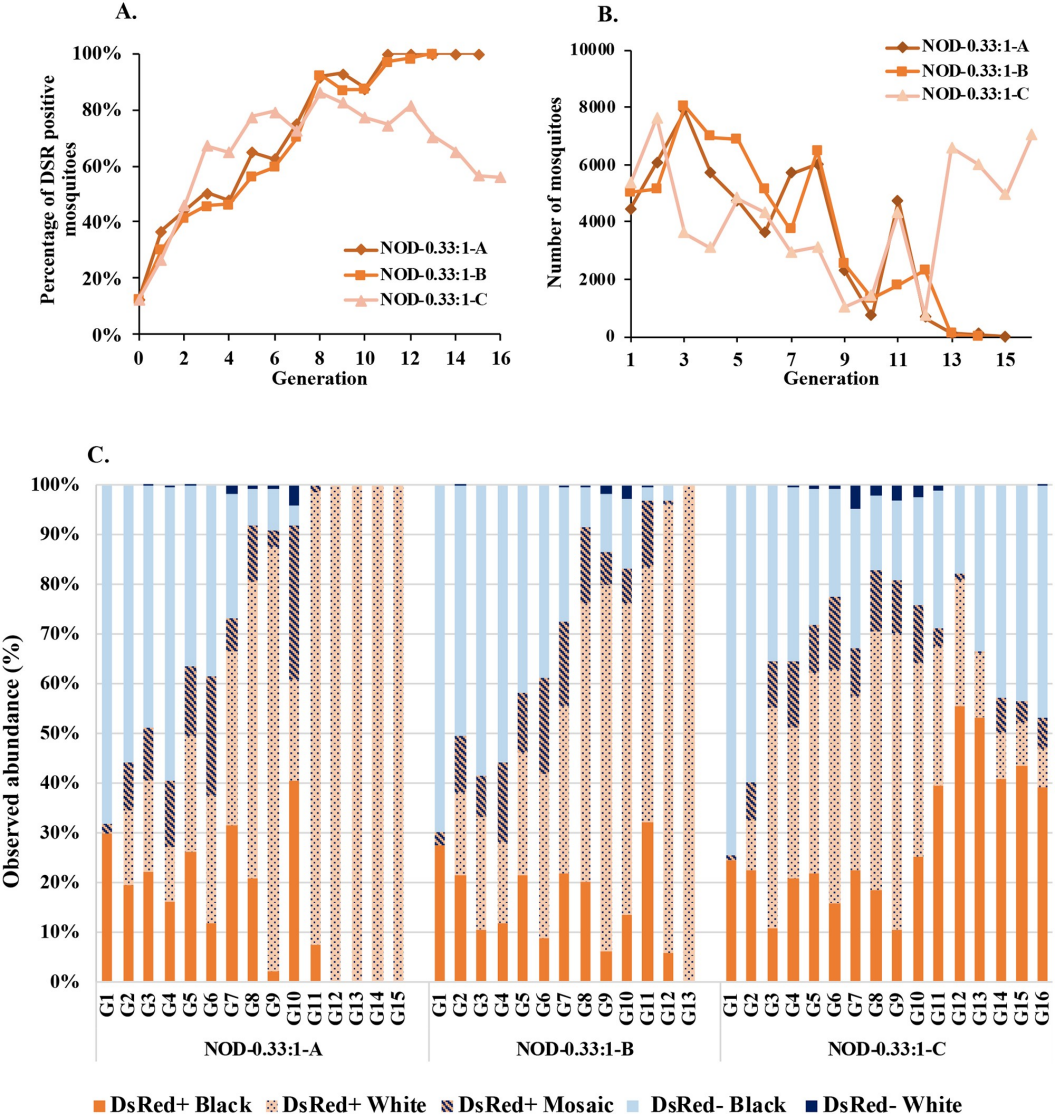
Non-overlapping gene drive (NOD) cage trial with initial releases of 0.33:1 transgenic AsMCRkh2 to wild-type males. Three cages (NOD-0.33:1A, NOD-0.33:1B and NOD-0.33:1) were seeded initially with 100 wild-type female, 75 wild-type male and 25 AsMCRkh2 male pupae. The resulting next generation larvae, pupae and adults were scored as DsRed-positive (DsRed^+^, carrying the AsMCRkh2 gene drive cassette) or wild-type. (A) Percentages of DsRed-positive (DsRed^+^) adult mosquitoes (Y-axis) in the total population in each cage scored at each generation (X-axis). (B) Total population size (Y-axis) in each replica cage at each generation (X-axis). (C) Abundance in percentages (Y-axis) of eye color and DsRed phenotypes of ~500 randomly-selected pupae per generation in each cage.

The percentages of first generation DsRed^+^ larvae in the 0.33:1 cages were 36% (NOD-0.33:1-A, 1569/4249), 30% (NOD-0.33:1-B, 1445/4818) and 26% (NOD-0.33:1-C, 1369/5134) (Fig 4; S19–S21 Tables). The expected percentage is 25% (1/4 chance of females mating with a transgenic male that experienced complete drive) if transgenic males are contributing equally with wild-type males to the next generation. Cages NOD-0.33:1-A and NOD-0.33:1-B had significantly higher contributions of transgenic males to the first generation (*Χ*^2^ = 242.04 [p = <0.00001] and 47.8 [p = <0.00001], respectively), whereas both types of males contributed equally in Cage NOD-0.33:1- C (*Χ*^2^ = 5.62 [p = 0.017]).

A rapid increase in DsRed^+^ mosquitoes follows immediately in the second generation of the 0.33:1 release ratio cages with full introduction in generations 12 and 13 in NOD-0.33:1-A and NOD-0.33:1-B, respectively (Fig 4, S19 and S20 Tables). Cage NOD-0.33:1-C, although initially having the highest percentage of DsRed^+^ mosquitoes from generations 2–6, did not go to full introduction, and reached 86% at its highest in generation 8 before it began to decrease. Cage NOD-0.33:1-C was terminated after generation 16 because molecular analyses described below of randomly-chosen samples of wild-type phenotype mosquitoes showed that there were no drive-sensitive wild-type alleles remaining in the population. As with the 1:1 release cages, population sizes fluctuated but dropped sharply in Cages NOD-0.33:1-A and NOD-0.33:1-B, and these went to extinction following full introduction of ablated *kh* alleles. Cage NOD-0.33:1-C nearly went extinct at generation 12 before recovering (Fig 4, S19 Table).

**Fig 5:**
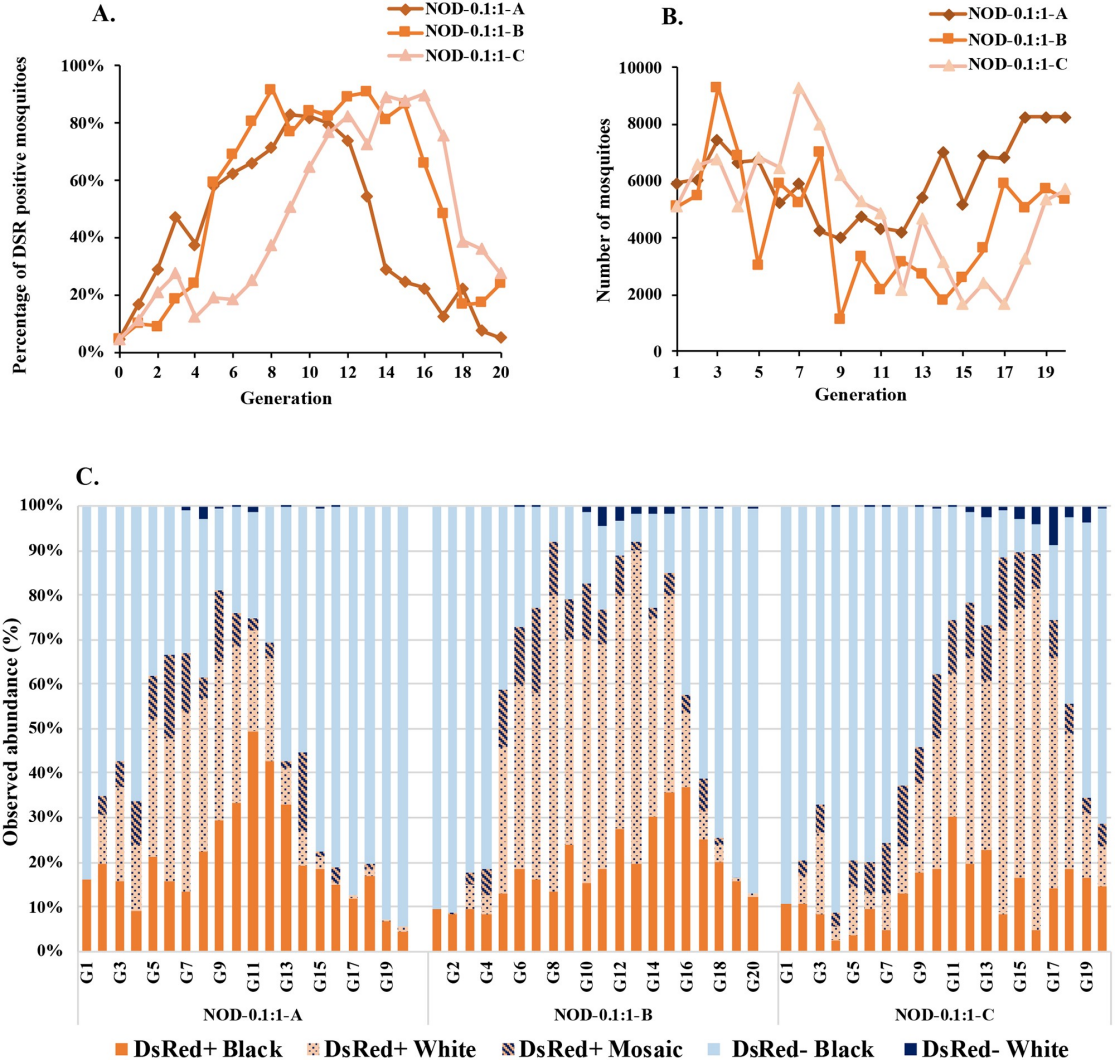
Non-overlapping gene drive (NOD) cage trial with initial releases of 0.1:1 transgenic AsMCRkh2 to wild-type males. Three cages (NOD-0.1:1A, NOD-0.1:1B and NOD-0.1:1) were seeded initially with 100 wild-type female, 90 wild-type male and 9 AsMCRkh2 male pupae. The resulting next generation larvae, pupae and adults were scored as DsRed-positive (DsRed^+^, carrying the AsMCRkh2 gene drive cassette) or wild-type. (A) Percentages of DsRed-positive (DsRed^+^) adult mosquitoes (Y-axis) in the total population in each cage scored at each generation (X-axis). (B) Total population size (Y-axis) in each replica cage at each generation (X-axis). (C) Abundance in percentages (Y-axis) of eye color and DsRed phenotypes of ~500 randomly-selected pupae per generation in each cage.

As with the previous overlapping generation experiments, the 0.1:1 release ratio cage populations never reached full introduction (Fig 5, S21 and S22 Tables). First-generation DsRed^+^ percentages were 17% (NOD-0.1:1-A, 985/5735), 10% (NOD-0.1:1-B, 509/4900) and 12% (NOD-0.1:1-C, 576/4896). With an expected percentage of ~10% if all transgenic males experience full drive, Cages NOD-0.1:1-A and NOD-0.1:1-C had significantly higher contributions of the transgenic than wild-type males to the first-generation progeny (*Χ*^2^ = 294 [p = <0000.1] and 15.09 [p = <0.0001] for cages NOD-0.1:1-A and NOD-0.1:1-C, respectively) and Cage NOD-0.1:1-B was equal (*Χ*^2^ = 0.736 [p = 0.39]).

The frequency of DsRed^+^ mosquitoes increased and reached a maximum of ~92% (Cage NOD-0.1:1-B, 6443/7005) at generation 8. Cage NOD-0.1:1-A followed with a maximum of 83% (3331/4012) at generation 9. Cage NOD-0.1:1-C lagged but also reached a maximum of 83% (2005/2427) at generation 16. These frequencies contrast with the results of the overlapping generation 0.1:1 drive trials in which the DsRed^+^ mosquitoes never were established.

All three 0.1:1 ratio cages showed rapid loss of DsRed^+^ mosquitoes as early as generation 9 (NOD-0.1:1-A) or after generation 16 (Cages NOD-0.1:1-B and NOD-0.1:1C), immediately after the respective maximum introduction of the transgenes. (Fig 5; S21 and S22 Tables). Similar to cage NOD-0.33:1-C, population size and the frequency of DsRed^+^ individuals in the population were related inversely due to the population suppression effect of the *kh* fitness impact.

### Molecular analysis of potential drive-resistant alleles in the cage populations

Amplified fragments of the target *kh* gene were sequenced from all adult mosquitoes with exceptional phenotypes (all white-eye only [DsRed^-^/*kh*^*w*^] and later-generation wild-type phenotype mosquitoes). The majority of white eye-only mosquitoes had small insertion or deletion (indel) mutations 1–20 base-pairs (bp) in length or non-synonymous substitutions at or near the cut site of the endonuclease, presumably arising from NHEJ (S23 Table). There were 50 independent NHEJ mutations arising among the 185 DsRed^-^/*kh*^*w*^ mosquitoes sampled from the nine cages; 10 of which had independent origins in more than one cage. The white-eye phenotypes resulted from both true homozygotes (isoallelic) and heteroallelic combinations.

A total of 12 distinct in-frame mutations were identified that resulted in disruption of KH enzymatic activity and a white-eye phenotype. All of these affected the codons for one or both of the amino acids, tyrosine (Y328) and glycine (G329), at the gRNA-directed cut site, 1179-TACGGG. Previous work has shown that a homozygous in-frame deletion of the tyrosine codon (TAC) and substitution of the adjacent glycine for a tryptophan (G329W) were sufficient to cause a white-eye phenotype [13]. A similar homozygous deletion of the glycine codon (GGG) also results in the same phenotype. One set of deletions arose independently in Cages NOD-1:1-A and NOD-0.33:1-A through recombination of the left homology arm with the short 20 nucleotide *kh*-targeting sequence encoded in the kh2 guide RNA (S23 Table).

**Fig 6:**
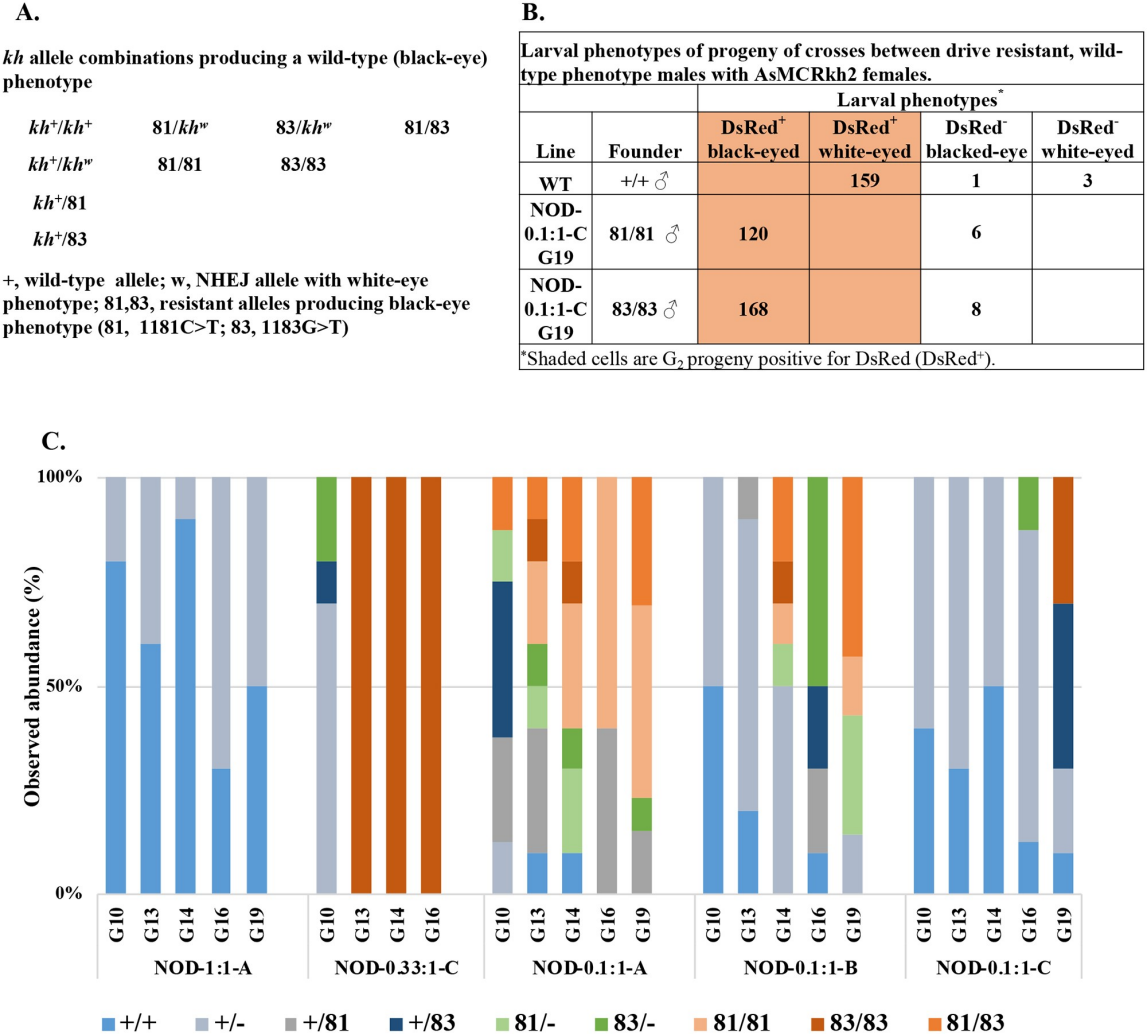
Wild-type *kh* drive-resistant genotypes and phenotypes at later generations of non-overlapping gene drive (NOD)cage trials. Randomly-selected samples of ~10 mosquitoes that had DsRed-negative (DsRed^-^)/ black-eyed (*kh^+^*) phenotypes from each cage were sequenced to analyze the kh2 target site. Two point mutations recovered as wild-type phenotypes were identified as 1181C>T (designated 81), which results in a silent mutation at Y328, and 1183G>C (designated as 83), which causes a substitution of its neighbor amino acid G329A. (A) Combinations of 81 and 83 alleles that result in wild-type phenotypes. (B) Larval phenotypes of progeny from crosses between DsRed^-^/black-eye (DsRed^-^/ *kh^+^*) individual mosquitoes homozygous for the drive-resistant alleles and homozygous AsMCRkh2 females (DsRed^+^/ *kh^w^*). (C) Percentages of each genotype present in each cage.

Molecular analysis of ~10 wild-type phenotype (DsRed negative, black eye) mosquitoes recovered from each NOD cage at later generations (G_10_, G_13_, G_14_, G_16_ and G_19_) showed that they had combinations of a functional wild-type alleles (*kh*^*+*^), silent mutations at Y328 (third position C to T transition) and substitutions of G329A (second position G to C transversion) (Fig 6, S6 Fig, S23 Table). Crossing mosquitoes homozygous for the two resistant alleles with AsMCRkh2 females showed complete inhibition of copying of a maternal drive allele into a paternal wild-type allele as all progeny had black eyes (Fig 6B). These ‘drive resistant’ mutations were recovered independently from four cages (NOD-0.33:1-C, NOD-0.1:1-A, NOD-0.1:1B and NOD-0.1:1-C) at different frequencies and in different combinations with wild-type and NHEJ alleles (Fig 6C). Importantly, the 1183G>C variant was present as homozygous in all wild-type phenotype mosquitoes sequenced in late-generation NOD-0.33:1-C cage samples. This allele configuration is the most likely explanation for the resurgence of the population in that cage. Cage NOD-1:1-A is difficult to interpret as the drive-system introduction stalls at generation 2 and remains at just over 60% through generation 10 (Fig 3). Modeling results, described below, suggest this could be due to a combination of stochasticity and/or resistant allele formation. After that, it fluctuates before starting to climb again at generation 18. Remarkably, no functional drive-resistant alleles were observed in the sequenced samples, although there were non-functional resistant alleles present as heterozygotes with wild-type alleles (Fig 6C).

### Modeling results

**Fig 7:**
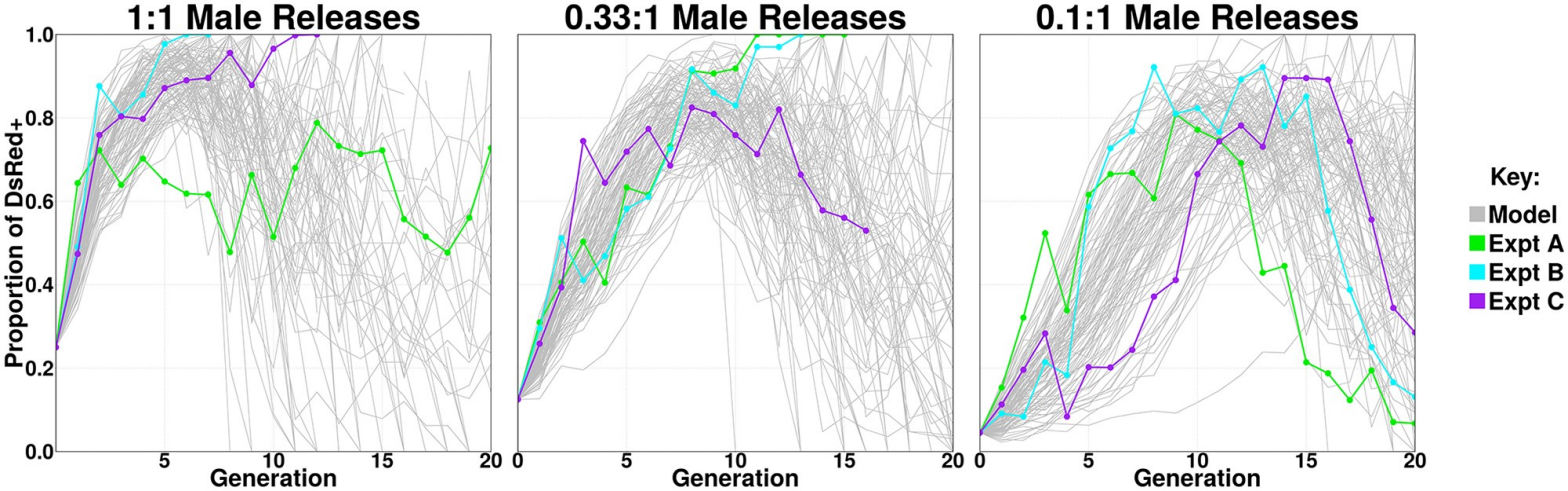
Observed and predicted dynamics with respect to the DsRed marker phenotype for non-overlapping experiments with the AsMCRkh2 gene drive system. Experimental data represent the triplicate replicates shown in Figs 3–5 and are displayed individually as green, blue and purple lines and connecting same-colored dots in each of the panels. Results from stochastic realizations of the fitted model (depicted in S2–S5 Files) are shown as thin grey lines (100 simulations per release ratio). Cages were set up with wild-type females and appropriate males to achieve 1:1, 0.33:1 and 0.1:1 (left, middle and right panels, respectively) transgenic gene-drive male release ratios. DsRed^+^ phenotype frequencies were monitored over 20 generations for the 1:1 and 0.1:1 releases, and over 17 generations for the 0.33:1 releases. The X-axis is the generation number after initial introduction and the Y-axis is the proportion of mosquitoes showing the DsRed marker phenotype (DsRed^+^). The DsRed^+^ phenotype results from having at least one copy of the gene-drive allele and hence reflects the spread of the gene drive system, sometimes to full introduction for 1:1 and 0.33:1 releases. The stochastic model captures the variability inherent in the experimental process and reflects the dual possibilities of gene drive full introduction and population reversion to a wild-type/drive-resistant state.

To characterize the population dynamics observed in the nine non-overlapping gene drive experiments, we fitted a mathematical model of autosomal CRISPR-based homing gene drive to the observed data. The model included two varieties of resistant alleles–an in-frame, cost-free resistant allele (R), and an out-of-frame or otherwise costly “broken” resistant allele (B)–genotype-specific fitness costs, and maternal deposition of Cas9 (see S1 Text for details). Through model fitting, the observed data were found to be consistent with homing efficiencies inferred from generation G_0_, namely an accurate homing efficiency of 95% in females and 98% in males, and with 0.5% (95% CrI: 0.0–3.6%) of resistant alleles being in-frame, cost-free (R), and the remainder being out-of-frame or otherwise costly (B). Maternal deposition of Cas9 was inferred to result in cleavage of embryonic W alleles with a frequency of 70% (95% CrI: 68–72%), with 22% (95% CrI: 21–24%) of the cleaved W alleles becoming R alleles, and the remainder becoming B alleles. Given these rates, the data are consistent with the following fitness costs: females homozygous for the homing and/or broken resistant allele (HH, HB or BB) are infertile, while the H, R and B alleles otherwise have multiplicative fitness costs per copy of 7.9% (95% CrI: 7.4–8.6%), 18.4% (95% CrI: 17.7–19.1%), and 0.0% (95% CrI: 0.0–0.0%), respectively. The resulting model fits are depicted in S1 Text (S2–S5 Files), with a stochastic implementation depicted in Fig 7.

Modeling results are consistent with a highly-efficient drive system capable of spreading to full introduction and inducing a population crash, but also with the emergence of in-frame resistant alleles capable of preventing the spread of the drive system, with either outcome possible by chance. Model predictions in S2–S5 Files depict how, for fitted parameters, the trajectory of DsRed^+^ individuals (i.e. those having at least one copy of the H allele) aligns well with the experiments in which the H allele spreads to full introduction, up to generation ~7–10. At this point, the dynamics of the fitted deterministic model are dominated by the spread of in-frame resistant and/or wild-type alleles (as reflected by the increase in frequency of the kh^+^ marker phenotype), and the model predictions align well with experiments in which the frequency of DsRed^-^ individuals begins to decline.

The stochastic model implementation captures chance events due to mate choice (multinomial-distributed), egg production (Poisson-distributed), offspring genotype (multinomial-distributed), and sampling of the next generation (multivariate hypergeometric-distributed). Incorporating stochasticity from each of these sources (see S1 Text for details), i) the H allele can spread resulting in a population crash (as seen in Cages NOD-1:1-B and C, and NOD-0.33:1-A and B), or ii) the H allele can decline and an in-frame resistant and/or wild-type allele can dominate (as seen in Cages NOD-1:1-A, NOD-0.33:1-C, and NOD-0.1:1-A, B and C). Temporary stagnation (as seen in Cage NOD-1:1-A) is also a possibility. Each of these outcomes is realized in the stochastic simulations with full introduction (and hence a population crash) occurring over the experimental period (20 generations) in 76% of simulated 1:1 releases (2 of 3 observed), 38% of simulated 0.33:1 releases (2 of 3 observed), and 14% of simulated 0.1:1 releases (0 of 3 observed) (Fig 7). When simulations were extended to 40 generations, the proportion that result in a crash increased to 87% for simulated 1:1 releases, 69% for simulated 0.33:1 releases, and 60% for simulated 0.1:1 releases. In 1% of the simulated 1:1 releases, the population did not crash; but the transgene was maintained in >90% of adult mosquitoes for >10 generations. This outcome was not observed for simulated 0.33:1 or 0.1:1 releases. Another possible outcome is that the population becomes fixed for resistant alleles. After 40 generations, this had happened in 7% of simulated 1:1 releases, 17% of simulated 0.33:1 releases, and 18% of simulated 0.1:1 releases.

## Discussion

We report here results from three different laboratory cage trial designs exploring population modification strains of *An*. *stephensi*. These were limited in scope to focus on the introduction of transgenes with and without a gene-drive component. Although the two different introduction modalities included transcriptionally-active scFv anti-malarial effector genes, parasite challenge assays were not part of the specific trial design as none of the strains tested here are proposed for further use as release strains. Parasite-resistance phenotypes are important and will be tested in future trials featuring next-generation population modification strains.

The three trials varied in the size of the cage (0.216 m^3^ vs 0.005 m^3^), whether or not the target population was age-structured, source of blood meal (mice or artificial feeder), number of releases (single or multiple) and whether the cage populations were maintained as overlapping or non-overlapping generations. In addition, release ratios varied among the trials with all sharing a 1:1 transgenic:wild-type male set of experiments.

A common feature of the trials is that all involved male-only releases. An early proposed design criterion for using genetically-engineered mosquitoes was that genes be introduced into populations by only releasing males [37]. It was thought that female releases would not be favored in trial communities because they would be nuisance biters. While there is modeling to support the release of gravid females because they produce clusters of progeny that amplify the release ratios and seek out favorable oviposition sites [38], we chose males-only for this series of laboratory cage trials. In addition, all males released were effectively homozygous for their respective transgenes. The AP26 males in the non-drive release were made so by intercrossing the line, and the AsMCRkh2 males had two copies of their transgene by virtue of the strong drive in their germline.

We expected these trials to provide preliminary information on possible genetic loads associated with the transgenes that would affect the performance of the modified strains. The results from the non-drive release and overlapping generation gene drive trials support the conclusion that there is no major load resulting from the integration of these transgenes that affects larval-to-adult survival. While there was variability among cages in survival, this was not biased greatly for either the transgenic or wild-type mosquitoes. The mosquitoes in all experiments were subjected to physical manipulations as they were moved from cages to microscopes for scoring, and this happened multiple times to individuals during the course of each replicate. Undoubtedly, there was an impact on survival from this manipulation, but it was spread across cages with transgenic insects subject to more handling as they were counted as larvae, pupae and adults.

Male mating success was measured indirectly by examining the contributions of introduced transgenic and wild-type males to first generation progeny. Since all gene-drive populations were established initially by adding pupae, variations in emergence times or similar effects that could cause either the wild-type or transgenic males to be present disproportionally should have affected first generations numbers. Furthermore, differences in sperm production, male aggressiveness and other factors also could bias the numbers. However, while transgenic and wild-type male contributions in both cage formats (0.005 and 0.216 m^3^) and in the overlapping and non-overlapping generation trial designs could show some bias, these favored neither male type. These results are consistent with previous work that shows that males from several transgenic lines did not suffer from the presence of inserted DNA [27, 31, 39]. However, we recognize that this is not to be expected of all transgenic mosquitoes and all types of transgene constructs. Insertion-site influences (mutagenesis, proximity of cis-regulatory DNA), expression characteristics of the transgenes and insertion copy number are likely to result in strains in which male mating competitiveness and other life parameters are reduced [40]. Direct and specific experiments must be conducted to verify male competitiveness for next-generation strains.

In contrast, female survival and fecundity were affected greatly in the AsMCRkh2 gene-drive lines. This results from disruptions of the target *kh* gene, which has a role in a biochemical pathway that provides important products to detoxify the blood meal [35, 41, 42]. Any combination of gene disruptions (insertion of drive elements or NHEJ alleles) that deprive the insects of the functional KH enzyme results in a severe phenotype evident following blood feeding. Indeed, this phenotype is sufficiently strong to cause some cage populations to go extinct once all *kh* genes in all females have been disrupted. This phenotype also imposed strong selection pressure on the cage populations and two distinct resistant genes encoding a functional KH enzyme emerged that rescued the lethality and permitted long-term survival of some cage populations.

It is not straightforward to compare the introduction rates among the different trial designs and formats. Complications include the fact that the non-drive protocol had repeated transgenic male releases and required replenishment of both wild-type males and females in the cages over the course of the experiments, whereas the gene drive trials involved only a single release. However, all but one of the cages in all the trials achieved high levels of transgene introduction at their most effective release ratios within eight generations of the first release. As expected, introduction rates were related directly to the release ratios. The 10:1 non-drive release had a fast trajectory to reach >80% introduction, achieving this within ~3 generations. Two of the three cages in the 1:1 releases in the non-overlapping generation gene-drive trial reached this level within the same period validating the expectation that gene drive may be more efficient than non-drive releases (single versus multiple releases) for transgene introduction. The overlapping gene drive protocol took 1–2 generations longer at their best to achieve the same levels as the non-overlapping trials with the latest cage to reach 80% at generation 7. This is likely due to mated wild-type females in the overlapping trials continuing to contribute to population complexity (mated females blood-fed once can survive three weeks or longer in our laboratory conditions [31]). Clearly, multiple releases and higher ratios of gene drive constructs can be expected to accelerate the introduction of the transgenes.

Transgene frequencies fluctuated after reaching their maxima in the 10:1 ratio non-drive release protocol but stayed higher than 80% throughout the balance of the trial. Modelling transmission dynamics can help determine whether this level of presence of the effector molecules is high enough to have an impact on malaria transmission [43, 44]. If deemed worthy of follow up, it should be possible to achieve higher introduction levels by increasing release ratios and/or frequency of release, but this would be expected to come with increased costs.

The overlapping gene-drive trial was designed originally to look only at the frequency of transgene increase following introduction and not carried out long enough to determine what happens once cages go to near full introduction for the transgenes or mutant *kh* alleles. In contrast, the non-overlapping gene drive trials produced two different outcomes, complete extinction of the cage population or loss of transgenes following maximum introduction. For example, two cages each in the 1:1 and 0.33:1 release trials went extinct with the former occurring at generations 8 and 13 and the latter at generations 14 and 16. The lag between the two sets of results likely reflects the initial release ratios. One cage each in the 1:1 and 0.33:1 trials started to decline in transgenes frequencies around generations 14 and 8, respectively, following a period when they both achieved >80% introduction. In the latter case, there was accumulations of drive-resistant NHEJ gRNA-target alleles. Modeling results are consistent with a highly efficient drive system capable of spreading to full introduction, but also with the emergence of in-frame resistant alleles capable of preventing spread, with either outcome possible by chance, including the stagnation of genotype frequencies seen in Cage NOD-1:1-A.

The primary sequence analysis of the NHEJ mutations resulting from the non-overlapping generation trial design revealed the anticipated array of insertions and deletions close to the gRNA-directed Cas9 cleavage site. These results are consistent with previous analyses in experiments with Anopheline mosquitoes and other flies [13, 14, 45–50]. One set of events was the independent recovery of crossovers between the left-hand genomic homology region and the short complementary sequence present in the synthetic gRNA encoded in the autonomous gene-drive element. Recombination within short sequences has been seen previously in mosquitoes, but these were associated with close tandem duplications of short (3 bp in length) sequences in the gRNAs [48]. We found no evidence of these events in our data, most likely because the kh2 gRNA design does not have any repetitive sequences encoded in it [13]. Future disruptions of the drive elements could be prevented by cloning the gRNA-generating sequence in the opposite orientation. Pairing and recombination at this locus then would lead to dicentric and acentric chromosomes and fragments that would be expected to be lethal at the cellular level.

One of the surprises from the NHEJ allele primary structure analysis was the large number of in-frame *kh* mutations that resulted in white-eye phenotypes. This is strong molecular evidence that the kh2 gRNA targets a region of the gene that is essential for its function. Our primary sequence analyses of the NHEJ and drive-resistant alleles identify a strong contribution of tyrosine 328 and glycine 329 to the function of the wild-type KH enzyme. The only tolerated variations were a drive-resistant synonymous mutation conserving the tyrosine residue or a glycine-to-alanine substitution. The *kh* gene and its orthologs in other mosquito species have been studied extensively and all produce a white-eye phenotype in homozygous mutant animals [13, 35, 51]. Indeed, rescue of a homozygous deletion of the gene in the yellow fever mosquito, *Aedes aegypti*, by the ortholog (*cinnabar*) from the fruit fly, *Drosophila melanogaster*, provided the visible marker for the development of the first transposon-based transgenesis systems in mosquitoes [52, 53]. Our inability to maintain a mutant homozygous *An*. *stephensi* line contrasts with what is seen in *Ae*. *aegypti* [54]. However, Yamamoto *et*. *al*. [51] were able to use a TALEN (transcriptional activator-like effector nuclease) system to generate a viable *kh* homozygous mutant *An*. *stephensi* line. The TALENs targeted a site different from the kh2 gRNA that we used, but this does not explain the differences we see as they also were able to make a deletion line that included the kh2 target sites. Differences in the mosquito midgut microbiome or other aspects of the feeding regimen may have supplied sufficient dietary supplementation to rescue the phenotype.

There is considerable discussion in the recent literature about the challenges to gene-drive technologies represented by guide RNA target sites that are potentially resistant to cleavage due to nucleotide variation [45–50]. These variants can be either naturally-occurring nucleotide polymorphisms or those induced by NHEJ following Cas9 mutagenesis of a previously-cleaved gRNA target site. Remarkably, empirical studies done in laboratory cages show different effects depending among other things on the species of insect and the targeting gRNA. Some studies show that NHEJ-induced polymorphisms in the target site could dampen drive dynamics, and in some cases, stop further introduction completely [45, 46, 48, 49]. This includes drive systems targeting female reproductive genes in mosquitoes and sex determination loci (*transformer*) in vinegar and fruit flies, all of which would be subject to strong positive selection. In contrast, a recent study to develop a population suppression strain for *An*. *gambiae* targeting the *doublesex* gene reports no selection of resistant alleles in their experiments [50]. Various ways to mitigate the impact of the NHEJ resistant alleles include using multiple guide RNA target sites, engineering dominant lethal allelic variants, limiting the size of the target population to be suppressed and inheritance patterns (through males or females only, or both sexes) [13, 46–48, 50]. Next generation designs of drive systems will have to find combinations of these mitigating features that work best for the specific target species.

The practical implications of resistant guide RNA target sites on population modification depend on which of the two alternatives represents the greatest source of resistance. If naturally-occurring SNPs are more frequent at the target site than Cas9-induced NHEJ alleles, then it is prudent to select another, less variable target site, especially if they are under positive selection [14]. If the frequency of natural resistant alleles was within a ‘tolerable’ range, and not expected to increase as a result of the drive machinery, then the challenge is to make sure the drive-induced alleles do not increase the frequency above that. Various gene drive modeling efforts have made initial estimates of what is tolerable variation, but these need further, more detailed analyses informed by empirical data with a prototype drive system [44, 47].

These preliminary cage trial experiments confirmed the potential feasibility of using a non-drive release approach to introgress anti-malaria effector genes into populations. This is not a trivial result because its success depends among other things on male mating competitiveness and female fertility and fecundity of the strain. The strain used here, AP26, does not seem to have any major genetic loads associated with it. However, it is important to emphasize that this strain (or any other of this type) has to be tested vigorously in parasite challenge assays that include variations in climate conditions and parasite loads and diversity [55–57]. It is likely that second-generation effector molecule cassettes will have to be developed and tested before this approach moves forward. Furthermore, success at introduction was only achieved with repeated 10:1 releases each week over the period of the experiment. The logistics of rearing and releasing that number of mosquitoes, for example in a major urban area, may be too costly and difficult to sustain. This is one of the reasons that gene-drive approaches have attracted so much interest [58]. Finally, non-drive releases may not be operationally sustainable. Cessation of a program is likely to lead to reverses as migrating wild-type mosquitoes lower transgene allele frequencies in the treated areas. Support and enthusiasm for continued long-term releases are likely to wane as malaria prevalence and incidence decrease, creating circumstances for a new epidemic.

The gene drive trials conducted here are essentially a variant of the ‘reduce and replace’ strategy modeled by Robert *et al*. (2013) [59]. Four cages in the non-overlapping generation trials went to extinction, highlighting the feasibility of this approach. However, strong selective pressures work to mitigate the genetic component responsible for population reduction, here evidenced by the increase in frequency of the drive-resistant NHEJ alleles that produce the functional KH enzyme. This also was seen in small cage trials of a suppression strategy for *An*. *gambiae* where resistant alleles restored female fertility [48].

Another interesting observation was that it was possible to get high levels of introduction, all above 80%, in all of the non-overlapping generation gene-drive trials with the 1:1 and 0.33:1 release ratios. The 1:1 ratio in the overlapping generation trials also went above 80%, but the 0.1:1 trials in this series failed to establish the transgene. Furthermore, the 0.1:1 releases in the non-overlapping experiments all achieved >90% DsRed^+^ mosquitoes before falling. The different outcomes of the two experiments may be an artifact of their experimental design resulting from how mosquitoes were either returned to their parental cages (overlapping) or were used to set up next generation cages (nonoverlapping). Since any wild populations are expected to comprise overlapping generations, these data support the interpretation that small releases of this specific strain, intentionally or inadvertently, may not be sufficient to instigate a population-wide sweep of the gene drive system, and that some practical threshold for release ratios may be needed to see complete gene introduction.

The concept of Target Product Profiles (TPP) was reviewed recently in the context of mosquito population modification approaches [10]. The key objective is to define how good the product has to be in order to be deployed in the field. Key to the further development of any strain, drive or non-drive, will be a clarification of what is minimally acceptable for a product to move forward. The types of cage trials described here can help provide empirical values to some of the TPP features. We were able to sample >600,000 mosquitoes over 20 generations in the small cage (0.005 m3) format and in-depth analysis of 500 of these from each cage at each generation allowed us to see the emergence of multiple types and frequencies of drive-resistant target sites. Both overlapping and non-overlapping generation trials designs provide preliminary estimates of male mating competitiveness. We conclude that a mix of overlapping and non-overlapping generation cage trials using transgenic lines carrying both dominant and, where possible, recessive marker genes can provide important information on gene drive parameters. What remains to be done is to add parasites challenge assays with multiple distinct parasite isolates.

## Supporting information

S1 Table Larval and adult phenotypes for non-drive release control cage ND-Control-A. (XLSX)

S2 Table Larval and adult phenotypes for non-drive release control cage ND-Control-B. (XLSX)

S3 Table Larval and adult phenotypes for non-drive release control cage ND-Control-C. (XLSX)

S4 Table Larval and adult phenotypes for non-drive release cage ND-1:1-A, 1:1 transgenic:wild-type male ratio. (XLSX)

S5 Table Larval and adult phenotypes for non-drive release cage ND-1:1-B, 1:1 transgenic:wild-type male ratio. (XLSX)

S6 Table Larval and adult phenotypes for non-drive release cage ND-1:1-C, 1:1 transgenic:wild-type male ratio. (XLSX)

S7 Table Larval and adult phenotypes for non-drive release cage ND-10:1-A, 10:1 transgenic:wild-type male ratio. (XLSX)

S8 Table Larval and adult phenotypes for non-drive release cage ND-10:1-B, 10:1 transgenic:wild-type ratio. (XLSX)

S9 Table Larval and adult phenotypes for non-drive release cage ND-10:1-C, 10:1 transgenic:wild-type male ratio. (XLSX)

S10 Table Larval and adult phenotypes for overlapping gene-drive cage OD-1:1-A, 1:1 AsMCRkh2:wild-type male release ratio. (XLSX)

S11 Table Larval and adult phenotypes for overlapping gene-drive cage OD-1:1-B, 1:1 AsMCRkh2:wild-type male release ratio. (XLSX)

S12 Table Larval and adult phenotypes for overlapping gene-drive cage OD-1:1-C, 1:1 AsMCRkh2:wild-type release ratio. (XLSX)

S13 Table Numbers of white-eyed and mosaic-eyed DsRed^+^ mosquitoes in overlapping gene-drive experiments. (XLSX)

S14 Table Larval and adult phenotypes for overlapping gene-drive cage OD-0.1:1-A, 0.1:1 AsMCRkh2:wild-type male release ratio. (XLSX)

S15 Table Larval and adult phenotypes for overlapping gene-drive cage OD-0.1:1-B, 0.1:1 AsMCRkh2:wild-type male release ratio. (XLSX)

S16 Table Larval and adult phenotypes for overlapping gene-drive cage OD-0.1:1-C, 0.1:1 AsMCRkh2:wild-type male release ratio. (XLSX)

S17 Table Total population size and larval DsRed phenotypes for non-overlapping gene-drive cages NOD-1:1 (A, B and C) with single release ratios of 1:1 AsMCRkh2:wild-type males. (XLSX)

S18 Table Pupal eye phenotypes for non-overlapping gene-drive cages NOD-1:1 (A, B and C) with the release ratios of AsMCRkh2:wild-type 1:1. (XLSX)

S19 Table Total population size and larval DsRed phenotypes for non-overlapping gene-drive cages NOD-0.33:1 (A, B and C) with single release ratios of 0.33:1 AsMCRkh2:wild-type males. (XLSX)

S20 Table Pupal eye phenotypes for non-overlapping gene-drive cages NOD-0.33:1 (A, B and C) with the release ratios of AsMCRkh2:wild-type 0.33:1. (XLSX)

S21 Table Total population size and larval DsRed phenotypes for non-overlapping gene-drive cages NOD-0.1:1 (A, B and C) with single release ratios of 0.1:1 AsMCRkh2:wild-type males. (XLSX)

S22 Table Pupal eye phenotypes for non-overlapping gene-drive cages NOD-0.1:1 (A, B and C) with the release ratios of AsMCRkh2:wild-type 0.1:1. (XLSX)

S23 Table NHEJ allele sequences in non-overlapping gene-drive cage trials. (PDF)

S1 Text Model design, parameters and fitting to cage data. This file describes the model design, parameters and some fitting to cage data.
(DOCX)

S1 Fig attP26. 10.1 and AP26 data. Characterization of the attp26 10.1 φC31 docking site line and AP26 dual effector line. A docking site line, attp26 10.1, carrying a *φC31 attP* nucleotide sequence for site‐specific integration, a gene encoding the cyan fluorescent protein under control of a 3xP3 gene promoter and enhancers, was generated using *piggyBac*‐mediated transformation and characterized following previously‐published protocols [31,32]. (A) Southern blot analysis of *Bam HI*‐digested attp26 10.1 genomic DNA probed with a ^32^P‐labelled gene amplification product derived from the cyan fluorescent protein (CFP) open reading frame. A single hybridizing fragment (arrow) shows that this is a single‐copy insertion into the *An*. *stephensi* genome. (B) Inverse polymerase chain reaction (IPCR) protocols were used to amplify a portion of the genomic DNA flanking the chromosomal insertion site of the *attP* docking site. The bolded TTAA represents the recognition site for *piggy-Bac*‐mediated transposition. (C) The nucleotide sequence of the IPCR amplicon was used to identify single scaffolds containing the sequence in each of the two versions of published *An*. *stephensi* genomes (scaffold 00077 from the Indian strain [60] and scaffold KB664547 from the SDA 500 strain [https://www.vectorbase.org]). (D) Schematic representation of the integrated transgenes in the dual effector line, AP26. The two single‐chain antibody constructs, Vg‐m2A10 and Cp‐m1C3, have been described [31]. They have been cloned between two *gypsy* sequences [26] and are flanked to one side by sites for *lox*‐mediated gene excision and the 3xP3‐DsRed marker gene. Integration of the construct into the docking site generates *attL* and *attR* sites. E) Reverse transcriptase polymerase chain reaction (RT‐PCR) analysis of the expression of the Vg‐m2A10 and Cp‐m1C3 transgenes following a bloodmeal. Gene‐specific primers were used to amplify samples of RNA prepared from males (M), and females at 0, 4, 12, 24 and 48 hours (h) after a blood meal. C is a control with no template. RP S26 is a control using primers complementary to the ribosomal protein small protein 26 [32].
(TIF)

S2 Fig Schematic of non-drive release protocol. Sixty wild-type male and female larvae were added to each of nine 0.216 m^3^ cages (image). Beginning week 3, females were provided a bloodmeal weekly and eggs were collected and hatched. Sixty larvae were randomly selected and returned to their respective cages weekly until week 8 to create an age-structured population in the cages. Beginning week 9, the nine cages were randomly assigned in triplicate as ND-Control-A, B and C’, ‘ND-1:1-A, B and C’ and ‘ND-10:1-A, B and C’ AP26:wild-type male release ratios replicate trials. ‘ND’ refers to ‘non-drive’, 1:1 and 10:1 refer to transgenic to wild-type male release ratios, and ‘A’, ‘B’ and ‘C’ refer to the individual cage replicates. Females were again provided a bloodmeal weekly, and eggs were collected, hatched, and reared to pupae. 30 male and 30 female wild- type pupae were added back to their cages. Cages ND-Control-A, B and C had no additional pupae added. Cages ND 1:1 (A, B and C) had an additional 30 transgenic AP26 male pupae added. Cages ND-10:1-(A, B and C) had an additional 300 transgenic AP26 male pupae added over three days. 300 larvae from each of the nine cages were selected randomly and screened for the DsRed marker. This procedure was repeatedly weekly until week 22.
(TIF)

S3 Fig Schematic of the overlapping gene-drive protocol. 120 wild-type males and 120 wild-type females were added to each of six 0.216 m3 cages (image). Cages OD-1:1 (A, B and C) with a 1:1 AsMCRkh2 male release ratio had an additional 120 transgenic AsMCRkh2 males added. Cages OD-0.1:1 (A, B and C) with a 0.1:1 male release ratio had an additional 12 transgenic AsMCRkh2 males added. Every 3 weeks for 7 generations, adult females were provided mice for bloodmeals and eggs were collected and hatched. 240 larvae were selected randomly and returned to their respective cages. No additional AsMCRkh2 males were added. 300 larvae were selected randomly and screened for the DsRed marker. They were later screened as pupae and adults for eye-color and sex.
(TIF)

S4 Fig Schematic of the non-overlapping gene-drive protocol. Nine small 0.005 m3 cages (image) were set up in triplicate according to their transgenic AsMCRkh2:wild-type male release ratios. Cages NOD-1:1 (A, B and C) with a 1:1 male release ratio had 100 wild-type females, 50 wild-type males, and 50 AsMCRkh2 males added. Cages NOD-0.33:1 (A, B and C) with a 0.33:1 male release ratio had 100 wild-type females, 75 wild-type males, and 25 AsMCRkh2 males added. Cages NOD-0.1:1 (A, B and C) with a 0.1:1 male release ratio had 100 wild-type females, 90 wild-type males, and 9 AsMCRkh2 males added. Females were provided a bloodmeal and eggs were collected and hatched. For Cages NOD-1:1 (A, B and C) and NOD-0.33:1 (A, B and C), 200 larvae were selected randomly and used to populate new cages, separate from that of their parents, for the next generation. An additional 500 larvae were selected randomly and reared to pupae, when they were screened for the DsRed marker and eye-color. The 500 pupae were then reared to adults and scored by sex. All remaining larvae were screened for the DsRed marker. This procedure was repeated every 3 weeks for 20 generations. For Cages NOD-0.1:1 (A, B and C) in generations 1–12, all larvae were scored for the DsRed marker and 200 larvae reflecting the existing transgene frequency were used to populate new cages. Beginning generation 13, Cages NOD-0.1:1 (A, B and C) were set up identically to Cages NOD-1:1 (A, B and C) and NOD-0.33:1 (A, B and C).
(TIF)

S5 Fig Larval and adult phenotypes for non-drive, overlapping gene-drive and overlapping gene-drive cage trials. Fluorescent and bright-field images of a larva, pupa and adult. Larvae were screened for the DsRed phenotype (DsRed^+^ and DsRed^-^). Pupae and adults were screened for the eye color phenotypes (black eye *kh*^*+*^, white eye *kh*^*w*^ and mosaic *kh*^*mosiac*^) and sex (♂ and ♀). The black arrow indicates a patch of colored-cells in the white background of the mosaic eye.
(TIF)

S6 Fig Frequency of accumulation of white-eye NHEJ alleles. White-eyed/DsRed^-^ phenotype mosquitoes contained two (same or different) NHEJ alleles that disrupted kynurenine hydroxylase enzymatic activity and resist endonuclease cutting and homing events. The allele frequency was calculated from the observed white-eyed/DsRed^-^ individuals from ~500 mosquitoes screened for eye color phenotype. The initial NHEJ allele frequency was ~ 5% per generation in all cages.
(TIF)

S7 Fig Mutated sequences at the kh2 target site in selected wild-type phenotype mosquitoes in later generations from the non-overlapping gene-drive cage trials. The top sequence is the wild-type reference sequence at the target kh2 site in control mosquitoes. The PAM sequence is in the red box, the gRNA targeted sequence is in the blue box, and the gRNA-directed cleavage site is indicated by a vertical thin white line. Sequencing randomly selected samples of black-eyed DsRed-negative mosquitoes from each cage revealed that there were two mutations that were homozygous in these mosquitoes. A point mutation of 1181C>T led to a silent mutation of Y328 while 1183G>C results in a substitution (G329A). Sequenced mosquitoes showed that some were homozygous for either mutation while others were heterozygous for both. These mutations conserved the kynurenine hydroxylase enzymatic activity while preventing endonuclease cutting.
(TIF)

S1 File Crosses representing the inheritance pattern of the AsMCRkh2 drive system. “H” denotes the autosomal AsMCRkh2 homing gene drive system, “W” denotes the wild-type allele targeted by the homing system, “R” denotes an in-frame, cost-free homing-resistant allele, and “B” denotes an out-of-frame or otherwise costly “broken” homing-resistant allele. Alleles segregate in a Mendelian fashion, with the exception of the W allele of HW heterozygotes. A proportion, *p*_*H*_ = 0.5(1 + *cp*_*HDR*_), of the gametes produced by HW heterozygotes are H alleles, where half are already H alleles, and a proportion, *c*, of the W alleles are cleaved, with a proportion, *p*_*HDR*_, of those being subject to accurate homology-directed repair (HDR) and becoming H alleles. The rate of HDR is sex-specific–i.e. there is a value, *p*_*HDR*,*F*_, in females, and a value, *p*_*HDR*,*F*_, in males. Of the cleaved W alleles that do not become H alleles, a proportion, *p*_*RES*_, become R alleles, while the remainder, 1 − *p*_*RES*_, become B alleles. I.e., a proportion, *p*_*R*_ = 0.5*c*(1 − *p*_*HDR*_)*p*_*RES*_, of the W alleles of HW heterozygotes become R alleles, while a proportion, *p*_*B*_ = 0.5*c*(1 − *p*_*HDR*_)(1 − *p*_*RES*_), become B alleles. Finally, a proportion, 1 − *c*, of W alleles are not cleaved, and hence the proportion of W gametes produced by HW heterozygotes is *p*_*W*_ = 0.5(1 − *c*). Subsequent to fertilization, and not depicted here, the effects of maternal deposition of Cas are accommodated. This can lead to the W allele of an offspring being cleaved if the mother has the H allele, allowing Cas to be deposited in the embryo. We consider cleavage to occur in a proportion, *p*_*MC*_, of these embryos, with a proportion, *p*_*MR*_, of the cleaved W alleles become R alleles, and the remainder, 1 − *p*_*MR*_, becoming B alleles. This, and the inheritance pattern depicted here, are described fully in the supplementary S1 Text.
(TIF)

S2 File Observed and predicted AsMCRkh2 gene drive dynamics (DsRed and kh marker phenotypes). Observed and predicted dynamics with respect to the DsRed and kh marker phenotypes for non-overlapping generation experiments with the AsMCRkh2 gene drive system. Experiments were set up with 100 wild-type (WW, where W represents the wild-type allele) females, and 100 or 99 males. For 1:1 releases (left), the initial condition is 50 transgenic males heterozygous for the drive system (HW, where H represents the homing-based drive system) and 50 WW males, for 0.33:1 releases (middle), the initial condition is 25 HW males and 75 WW males, and for 0.1:1 releases (right), the initial condition is 9 HW males and 90 WW males. Population counts were monitored over 21 generations for the 1:1 and 0.1:1 releases, and over 17 generations for the 0.33:1 releases. Results from these experiments are shown as solid lines (3 experiments per release ratio), with fitted model predictions shown as dashed lines (1 simulation per release ratio). Observed data are consistent with homing efficiencies inferred from generation G_0_, namely an accurate homing efficiency of 95% in females and 98% in males, and with 0.5% (95% CrI: 0.0–3.6%) of resistant alleles being in-frame, cost-free (R), and the remainder being out-of-frame or otherwise costly resistant “broken” alleles (B). Furthermore, maternal deposition of Cas is inferred to result in cleavage of embryonic W alleles with a frequency of 70% (95% CrI: 68–72%), with 22% (95% CrI: 21–24%) of the cleaved W alleles becoming R alleles, and the remainder becoming B alleles. Given these rates, the data are consistent with the following fitness costs: females having two copies of the homing and/or broken resistant allele (HH, HB or BB) are infertile, otherwise the H, R and B alleles have multiplicative fitness costs per copy of 7.9% (95% CrI: 7.4–8.6%), 18.4% (95% CrI: 17.7–19.1%), and 0.0% (95% CrI: 0.0–0.0%), respectively. The DsRed^+^ phenotype is associated with having at least one copy of the gene drive allele (i.e. genotypes HH, HR, HB and HW), and hence reflects the spread of the gene drive system, sometimes to fixation for 1:1 and 0.33:1 releases. The *kh^+^* phenotype is associated with having at least one copy of the wild-type allele or in-frame drive-resistant allele (i.e. genotypes WW, RW, BW, HW, RR, RB and HR), and hence reflects the initial loss of the wild-type allele and subsequent spread of the in-frame resistant and/or wild-type allele. In the 1:1 and 0.33:1 releases where the drive allele spreads to full introduction, the *kh^+^* phenotype is lost.
(TIF)

S3 File Observed and predicted AsMCRkh2 gene drive dynamics (1:1 releases). Observed and predicted dynamics with respect to DsRed and *kh* marker phenotype combinations for 1:1 non-overlapping generation experiments with the AsMCRkh2 gene drive system. Experiments were set up with 100 wild-type (WW, where W represents the wild-type allele) females, 50 transgenic males heterozygous for the drive system (HW, where H represents the homing-based drive system) and 50 WW males. Population counts were monitored over 21 generations for experiment 1, although the population crashed earlier than this for experiments 2 and 3. Results from these experiments are shown as solid lines, with fitted model predictions as dashed lines. Model predictions are based on data from all 9 experiments (3 for each release ratio: 1:1, 0.33:1 and 0.1:1 HW:WW males), with estimated and inferred parameter values described in the Results section of the manuscript. DsRed^+^/kh^-^ individuals have the gene drive (H) allele, but not the wild-type (W) or in-frame resistant (R) allele (i.e. genotypes HH and HB). These genotypes spread to fixation in experiments 2 and 3, but stagnate in experiment 1. DsRed^-^/kh^-^ individuals lack both the H allele and the W or R allele (i.e. genotype BB). This genotype persists at low levels due to being consistently generated, but conferring infertility in females. DsRed^+^/*kh^+^* individuals have both the H allele and the W or R allele (i.e. genotypes HW and HR), and DsRed^-^/*kh^+^* individuals have the W or R allele, but lack the H allele (i.e. genotypes WW, RW, BW, RR and RB). In both cases, these genotypes persist in experiment 1; but are eliminated in experiments 2 and 3 as the H and/or B alleles spread to fixation.
(TIF)

S4 File Observed and predicted AsMCRkh2 gene drive dynamics (0.33:1 releases). Observed and predicted dynamics with respect to DsRed and *kh* marker phenotype combinations for 0.33:1 non-overlapping generation experiments with the AsMCRkh2 gene drive system. Experiments were set up with 100 wild-type (WW, where W represents the wild-type allele) females, 25 transgenic males heterozygous for the drive system (HW, where H represents the homing-based drive system) and 75 WW males. Population counts were monitored over 17 generations. Results from these experiments are shown as solid lines, with fitted model predictions as dashed lines. Model predictions are based on data from all 9 experiments (3 for each release ratio: 1:1, 0.33:1 and 0.1:1 HW:WW males), with estimated and inferred parameter values described in the Results section of the manuscript. DsRed^+^/kh^-^ individuals have the gene drive (H) allele, but not the wild-type (W) or in-frame resistant (R) allele (i.e. genotypes HH and HB). These genotypes spread to fixation in experiments 1 and 2, but stagnate in experiment 3. DsRed^-^/*kh^-^* individuals lack both the H allele and the W or R allele (i.e. genotype BB). This genotype persists at low levels due to being consistently generated, but conferring infertility in females. DsRed^+^/*kh^+^* individuals have both the H allele and the W or R allele (i.e. genotypes HW and HR), and DsRed^-^/*kh^+^* individuals have the W or R allele, but lack the H allele (i.e. genotypes WW, RW, BW, RR and RB). These genotypes persist in experiment 3; but are eliminated in experiments 1 and 2 as the H and/or B alleles spread to fixation.
(TIF)

S5 File Observed and predicted AsMCRkh2 gene drive dynamics (0.1:1 releases). Observed and predicted dynamics with respect to DsRed and kh marker phenotype combinations for 0.1:1 non-overlapping generation experiments with the AsMCRkh2 gene drive system. Experiments were set up with 100 wild-type (WW, where W represents the wild-type allele) females, 9 transgenic males heterozygous for the drive system (HW, where H represents the homing-based drive system) and 90 WW males. Population counts were monitored over 21 generations. Results from these experiments are shown as solid lines, with fitted model predictions as dashed lines. Model predictions are based on data from all 9 experiments (3 for each release ratio: 1:1, 0.33:1 and 0.1:1 HW:WW males), with estimated and inferred parameter values described in the Results section of the manuscript. DsRed^+^/kh^-^ individuals have the gene drive (H) allele, but not the wild-type (W) or in-frame resistant (R) allele (i.e. genotypes HH and HB). These genotypes spread to ~40–80% in the experiments, and to ~55% in the simulation, before declining. DsRed^-^/*kh^-^* individuals lack both the H allele and the W or R allele (i.e. genotype BB). This genotype persists at low levels due to being consistently generated, but conferring infertility in females. DsRed^-^/*kh^+^* individuals have the W or R allele, but lack the H allele (i.e. genotypes WW, RW, BW, RR and RB). These genotypes decline in frequency initially, and then begin to rise again beginning in generations 12–17, suggesting a resurgence of the R and/or W allele. Perhaps as a consequence of this, DsRed^+^/*kh^+^* individuals having both the H allele and the W or R allele (i.e. genotypes HW and HR) persist for the duration of the experiments.
(TIF)
