# Supplementary material for: Experimental population modification of the malaria vector mosquito, Anopheles stephensi
Source: PLoS Genet. 2019 Dec 19;15(12):e1008440. doi: 10.1371/journal.pgen.1008440 (PMC6922335; doi:10.1371/journal.pgen.1008440)
Supplement: S23 Table — (PDF) [file pgen.1008440.s023.pdf]

Table S23: NHEJ allele sequences in non-overlapping gene-drive cage trials

WT	GCGGCGCACGCGATGGTTCCGTTCTACGGGCAGGGCATGAACGCGGGCTTTGAAGACTGTAGC	Frame	Hom	Het
A1-G3	GCGGCGCACGCGATGGTTCCGTTCTACAGGGGCAGGGCATGAACGCGGGCTTTGAAGACTGTAGC	FS	1/2	
	GCGGCGCACGCGATGGTTCCGTTCTACGAGGCAGGGCATGAACGCGGGCTTTGAAGACTGTAGC*	FS		1/2
	GCGGCGCACGCGATGGTTCCGTTCTACGGAACAGGGCAGGGCATGAACGCGGGCTTTGAAGACTGTAGC	IF		1/2
A1-G7	GCGGCGCACGCGATGGTTCCG--------GCAGGGCATGAACGCGGGCTTTGAAGACTGTAGC	FS	5/7	
	GCGGCGCACGCGATGGTTCCGTTCTACG(ins473bp)GGCAGGGCATGAACGCGGGCTTTGAAGACTGTAGC*,++	FS	2/7	
A1-G10	GCGGCGCACGCGATGAAC-----------GCAGGGCATGAACGCGGGCTTTGAAGACTGTAGC	FS	6/8	1/8
	GCGGCGCACGCGATGGTTCCGTTCCC---GCAGGGCATGAACGCGGGCTTTGAAGACTGTAGC	IF	1/8	
A1-G13	GCGGCGCACGCGATGGTTCCGTTCTACG(ins473bp)GGCAGGGCATGAACGCGGGCTTTGAAGACTGTAGC*,++	FS		6/9
	GCGGCGCACGCGATGAAC-----------GCAGGGCATGAACGCGGGCTTTGAAGACTGTAGC	FS		5/9
A3-G5	GCGGCGCACGCGATGGTTCCGTTCTACAGGAACGTTCAT-GGCAGGGCATGAACGCGGGCTTTGAAGACTGTAGC	FS	1/4	1/4
	GCGGCGCACGCGATGGTTCCGTTCTACCATCATCAGGGCAGGGCATGAACGCGGGCTTTGAAGACTGTAGC	FS	2/4	1/4
A3-G6	GCGGCGCACGCGATGGTTCCGTTCTGGCCGTGGCAGGGCATGAACGCGGGCTTTGAAGACTGTAGC	FS		1/1
	GCGGCGCACGCGATGGTTCCGTTCTACGAGGCAGGGCATGAACGCGGGCTTTGAAGACTGTAGC*	FS		1/1
A3-G7	GCGGCGCACGCGATGGTTCCGTTCTACCATCATCAGGGCAGGGCATGAACGCGGGCTTTGAAGACTGTAGC	FS		1/1
	GCGGCGCACGCGATGGTTCCGTTCTACAAGGGAACAGGGCAGGGCATGAACGCGGGCTTTGAAGACTGTAGC	FS		1/1
A3-G7	GCGGCGCACGCGATGGTTCCGTTCTACGAGGCAGGGCATGAACGCGGGCTTTGAAGACTGTAGC	FS		5/5
	GCGGCGCAATCATCGGATCATCGGA--------------------------------GTAGC	FS		5/5
B1-G4	GCGGCGCACGCGATGGTTCCGTTCTACG(ins473bp)GGCAGGGCATGAACGCGGGCTTTGAAGACTGTAGC*,++	FS	2/3	
	GCGGCGCACGCGATGGTTCCGTTCTACA------GCATGAACGCGGGCTTTGAAGACTGTAGC	IF	1/3	
B1-G5	GCGGCGCACGCGATGGTTCCGTTCTACG(ins473bp)GGCAGGGCATGAACGCGGGCTTTGAAGACTGTAGC	FS	1/1	
B1-G7	GCGGCGCACGCGATGGTTCCGTTCTAC----AGGGCATGAACGCGGGCTTTGAAGACTGTAGC*	FS		9/9
	GCGGCGCACGCGATGGTTCCGTTCTACAGGGCAGGGCATGAACGCGGGCTTTGAAGACTGTAGC	FS		2/9
	GCGGCGCACGCGATGGTTCCGTTCTACGATGAACGATGGCAGGGCATGAACGCGGGCTTTGAAGACTGTAGC	IF		3/9
	GCGGCGCACGCGATGGTTCCGTTCTACGTGAACGATGGCAGGGCATGAACGCGGGCTTTGAAGACTGTAGC	FS		1/9
	GCGGCATGCCCTGC----------------------GGCAGGGCATGAACGCGGGCTTTGAAGACTGTAGC*	FS		3/9
B1-G10	GCGGCGCACGCGATGGTTCCGTTCTAC----AGGGCATGAACGCGGGCTTTGAAGACTGTAGC	FS	16/16	
B2-G7	GCGGCATGCCCTGC----------------------GGCAGGGCATGAACGCGGGCTTTGAAGACTGTAGC*	FS	2/2	
B2-G10	GCGGCGCACGCGATGGTTCCGTTCC---GGCAGGGCATGAACGCGGGCTTTGAAGACTGTAGC*	IF		12/14
	GCGGCGCACGCGATGGTTCCGTTCTGTTCCGGCAGGGCATGAACGCGGGCTTTGAAGACTGTAGC	FS		10/14
	GCGGCGCACGCGATGGTTCCGTTCT-CGGGCAGGGCATGAACGCGGGCTTTGAAGACTGTAGC	FS		2/14
	GCGGCGCACGCGATGGTTCCGTTCTACGAGGGCAGGGCATGAACGCGGGCTTTGAAGACTGTAGC	FS		2/14
	GCGGCGCACGCGATGGTTCCGTTC----<br>CGTTCCAACGGAACCCGGCAGGGCATGAACGCGGGCTTTGAAGACTGTAGC	IF		2/14
B2-G11	GCGGCGCACGCGATGGTTCCGTTCTACAGGAACGGAACCC--GCAGGGCATGAACGCGGGCTTTGAAGACTGTAGC	FS	1/2	1/2
	GCGGCGCACGCGATGGTTCCGTTCTA--GGCAGGGCATGAACGCGGGCTTTGAAGACTGTAGC	FS		1/2
B3-G4	GCGGCGCACGCGATGGTTCCGTTCTAC----AGGGCATGAACGCGGGCTTTGAAGACTGTAGC*	FS	1/2	
B3-G5	GCGGCGCACGCGATGGTTCCGTTCTACAGTACA-GGCAGGGCATGAACGCGGGCTTTGAAGACTGTAGC	FS	4/4	
B3-G6	GCGGCGCACGCGATGGTTCCGTTCTACAGTACA-GGCAGGGCATGAACGCGGGCTTTGAAGACTGTAGC	FS		4/4
	GCGGCGCACGCGATGGTTCCGTTCTACGTTCGCGGCAGGGCATGAACGCGGGCTTTGAAGACTGTAGC	FS		4/4
B3-G7	GCGGCGCACGCGATGGTTCCGTTCTACAGTACA-GGCAGGGCATGAACGCGGGCTTTGAAGACTGTAGC	FS		10/23
	GCGGCGCACGCGATGGTTCCGTTCTATGGCACGCGATGGTTCCGAT--<br>GGCAGGGCATGAACGCGGGCTTTGAAGACTGTAGC	FS		9/23
	GCGGCGCACGCGATGGTTCCGTTCC---GGCAGGGCATGAACGCGGGGCTTTGAAGACTGTAGC*	IF	2/23	16/23
	GCGGCGCACGCGATGGTTCCGTTCTACATGAACGC-GGCAGGGCATGAACGCGGGCTTTGAAGACTGTAGC	FS		2/23
	GCGGCGCACGCGATGGTTCCGTTCTACATTCCA-GGCAGGGCATGAACGCGGGCTTTGAAGACTGTAGC	FS		1/23
	GCGGCGCACGCGATGGTTCCGTTCTAC----AGGGCATGAACGCGGGCTTTGAAGACTGTAGC	FS		3/23
	GCGGCGCACGCGATGGTTCCGTTCATGCGGCAGGGCATGAACGCGGGCTTTGAAGACTGTAGC	IF		1/23
B3-G10	GCGGCGCACGCGATGGTTCCGTTCC---GGCAGGGCATGAACGCGGGGCTTTGAAGACTGTAGC	IF	2/12	3/12
	GCGGCGCACGCGATGGTTCCGTTCTAC-----GGGCATGAACGCGGGCTTTGAAGACTGTAGC	FS		6/12
	GCGGCGCACGCGATGGTTCCGTTCTACATTCGC-GGCAGGGCATGAACGCGGGCTTTGAAGACTGTAGC	FS		6/12
	GCGGCGCACGCGATGAAC--------------GCAGGGCATGAACGCGGGCTTTGAAGACTGTAGC	FS	1/12	
	GCGGCGCACGCGATGGTTCCGTTCTACGGTTCGC-GCAGGGCATGAACGCGGGCTTTGAAGACTGTAGC	FS		3/12
C1-G7	GCGGCGCACGCGATGGTTCCG--------GCAGGGCATGAACGCGGGCTTTGAAGACTGTAGC	FS	1/4	
C1-G11	GCGGCGCACGCGATGGTTCCGTTC----GGCAGGGCATGAACGCGGGCTTTGAAGACTGTAGC	FS	4/7	
C1-G13	GCGGCGCACGCGATGGTTCCGTTCTAC----AGGGCATGAACGCGGGCTTTGAAGACTGTAGC*	FS		1/3
	GCGGCGCACGCGATGGTTCCGTTCTA--GGCAGGGCATGAACGCGGGCTTTGAAGACTGTAGC	FS	1/3	1/3

	GCGGCGCACGCGATGGTTCCGTTC-------AGGGCATGAACGCGGGCTTTGAAGACTGTAGC	FS		1/3
	GCGGCGCACGCGATGGTTCCGTTCTACCAT----------ACGCGGGCTTTGAAGACTGTAGC	FS		1/3
C2-G7	GCGGCGCACGCGATGGTTCCGTTCTACTTTCGGGCAGGGCATGAACGCGGGCTTTGAAGACTGTAGC	FS	1/1	
C2-G10	GCGGCGCACGCGATGGTTCCGTTCTA--GGCAGGGCATGAACGCGGGCTTTGAAGACTGTAGC	FS	4/4	
C2-G13	GCGGCGCACGCGATGGTTCCGTTCTAC----AGGGCATGAACGCGGGCTTTGAAGACTGTAGC*	FS	1/9	1/9
	GCGGCGCACGCGATGGTTCCGTTCTACG-CATGCAGGGCATGAACGCGGGCTTTGAAGACTGTAGC	FS	2/9	6/9
	GCGGCGCACGCGATGGTTCCGTTCTACCAT----------ACGCGGGCTTTGAAGACTGTAGC	FS		1/9
	GCGGCGCACGCGATGGTTCCGTTCTACGGGCA--GCATGAACGCGGGCTTTGAAGACTGTAGC	FS		1/9
	GCGGCGCACGCGATGGTTCCGTTCTACGCAACCAGGCAGGGCATGAACGCGGGCTTTGAAGACTGTAGC	IF		
C3-G4	GCGGCGCACGCGATG-------------GGCAGGGCATGAACGCGGGCTTTGAAGACTGTAGC	FS		1/1
	GCGGCGCACGCGATGGTTCCGTTCTACAGGAACGGAACCC--GCAGGGCATGAACGCGGGCTTTGAAGACTGTAGC	FS		1/1
C3-G6	GCGGCGCACGCGATGGTTCCGTTCTAC----AGGGCATGAACGCGGGCTTTGAAGACTGTAGC	FS		1/1
	GCGGCGCACGCGATGGTTCCGTTCTAC-TCAGGGGCAGAACGGCAGGGCATGAACGCG	IF		1/1
C3-G7	GCGGCGCACGCGATGGTTCCGTTCTAC-GGCAGGGCATGAACGCG	FS		1/1
	GCGGCGCACGCGATGGTTCCGTTCA------GGGCATGAACGCG	IF		1/1
C3-G10	GCGGCGCACGCGATGGTTCCGTTCTACGAGGCAGGGCATGAACGCGGGCTTTGAAGACTGTAGC*	FS		
	GCGGCGCACGCGATGGTTCCGTTCTAC----AGGGCATGAACGCGGGCTTTGAAGACTGTAGC	FS		1/1
C3-G13	GCGGCGCACGCGATGGTTCCGTTCTAC---CAGGGCATGAACGCGGGCTTTGAAGACTGTAGC	IF	4/12	
	GCGGCGCACGCGATGGTTCCGTTCTA---GCAGGGCATGAACGCGGGCTTTGAAGACTGTAGC	IF		2/12
	GCGGCGCACGCGATGGTTCCGTTCC---GGCAGGGCATGAACGCGGGCTTTGAAGACTGTAGC*	IF		2/12
C3-G13	GCGGCGCACGCGATGGTTCCGTTCTACGAGGCAGGGCATGAACGCGGGCTTTGAAGACTGTAGC*	FS		2/12
C3-G16	GCGGCGCACGCGATGGTTCCGTTCTAC---CAGGGCATGAACGCGGGCTTTGAAGACTGTAGC	IF	1/12	4/12
	GCGGCGCACGCGATGGTTCCGTTCT---GGCAGGGCATGAACGCGGGCTTTGAAGACTGTAGC	IF		5/12
	GCGGCGCACGCGATGGTTCCGTTCTCC-GGCAGGGCATGAACGCGGGCTTTGAAGACTGTAGC	FS		4/12
	GCGGCGCACGCGATGGGCAGAC-------------CATGAACGCGGGCTTTGAAGACTGTAGC	FS		5/12

gRNA target site; PAM; insertion; substitution; deletion; *mutation appeared in multiple cages

++ : insertion sequence is from gRNA scaffold-U6-3'UTR

GGCGCACGCGATGGTTCCGTTCTACGGGCGTTTTAGAGCTAGAAATAGCAAGTTAAAATAAGGCTAGTCCGTTATCAACTTGAAAAGTGGCACCGAGTCGGTGCTTTTTT
TGTGGAAATTTGATTCACTTGTTTTAGGAAAATAATTACCTTCCTTTGAAACAGGTATTTACAATGATGGACGATAGAAAAAGACCACCTTAAAATGCTTTATTCAAGCT
ATTGCTTTCGATTCTTCATTGAGATCTAATCTCAAATTTGTGTATTTAGAAAAAACAGCGAATATTGTACTATAATAATTCTTTGGTAATCTCAGGCTTTCAGTAATGAT
AGAATCCTTAAGACTGTAAGAATATACAAGTTTTAAAAAAAGGAAAACAGGTATATCAAATCAGAAAACAACAACAGCTCTTTTCCGTTGAATTCTACCTGTACGATGGC
TTAAGGATGAGCTGTAACTGAAACGAAACCTTACAACAGCCACGCAGAACGGGATCCGGCAGGGCATGAACGC
