# Supplementary material for: Experimental population modification of the malaria vector mosquito, Anopheles stephensi
Source: PLoS Genet. 2019 Dec 19;15(12):e1008440. doi: 10.1371/journal.pgen.1008440 (PMC6922335; doi:10.1371/journal.pgen.1008440)
Supplement: S1 Text — This file describes the model design, parameters and some fitting to cage data. (DOCX) [file pgen.1008440.s024.docx]

**Smod: Model design, parameters and fitting to cage data.**

**Model Fitting.**

We modeled the population dynamics of the AsMCRkh2 gene drive system under laboratory cage conditions (non-overlapping generations) assuming discrete generations and a randomly mixing population. Here, we first describe the model fitting process. We then describe a stochastic implementation of the model allowing us to calculate the expected frequency of gene drive-induced population extinctions for different introduction frequencies.

To model the AsMCRkh2 system, we consider an autosomal homing allele (denoted by “H”), a wild-type allele (denoted by “W”), an in-frame, cost-free resistant allele (denoted by “R”), and an out-of-frame or otherwise costly resistant “broken” allele (denoted by “B”). This leads to 10 possible female and male genotypes (HH, HR, HB, HW, RR, RB, RW, BB, BW, and WW). We denote the proportion of organisms having each genotype at the $k$th generation by $p_{k}^{x,y}$, where $x$ denotes one of the 10 genotypes, and $y$ denotes the sex (“F” for female, and “M” for male).

Given the large number of possible mating pairs (100), it is not feasible to show the complete equations for the next generation genotype frequencies here, so we instead depict them in Fig. Smod S1, and describe them here in brief. We consider Mendelian inheritance rules at the gene drive locus, with the exception that, for HW heterozygotes, a proportion, $c$, of the W alleles are cleaved, while a proportion, $1-c$, remain as W alleles. Of those that are cleaved, a proportion, $p_{HDR}$, are subject to accurate homology-directed repair (HDR) and become H alleles, while a proportion, ${1-p}_{HDR}$, become resistant alleles. Of those that become resistant alleles, a proportion, $p_{RES}$, of these become in-frame, cost-free resistant (R) alleles, while the remainder, ${1-p}_{RES}$, become out-of-frame or otherwise costly resistant (broken, B) alleles. The resulting alleles segregate in a Mendelian fashion.

The effects of maternal deposition of Cas were accommodated after computing the gene drive-modified Mendelian inheritance rules. If offspring having a W allele had a mother having the H allele, then this would lead to Cas being deposited in the embryo by the mother, possibly resulting in cleavage of the W allele. We considered cleavage to occur in a proportion, $p_{MC}$, of these embryos, with a proportion, $p_{MR}$, of the cleaved W alleles become R alleles, and the remainder, ${1-p}_{MR}$, becoming B alleles.

These considerations allow us to calculate the expected genotype frequencies in the next generation before accounting for fitness costs. Let us denote these frequencies by $\hat{p}_{k+1}^{x,y}$, where $x$ denotes the genotype, $y$ denotes the sex, and $k+1$ denotes the next generation. Normalizing these ratios to account for fitness costs – multiplicative costs $s_{H}$, $s_{R}$ and $s_{B}$ associated with each copy of the H, R and B allele, respectively, and infertility of females having two copies of the H and/or B allele, $s_{HH,F}=1$, the genotype frequencies in the next generation are given by,

($p_{k+1}^{HH,F},p_{k+1}^{HB,F},p_{k+1}^{BB,F})=(\hat{p}_{k+1}^{HH},\hat{p}_{k+1}^{HB},\hat{p}_{k+1}^{BB})(1-s_{HH,F})/W_{k+1}$

$p_{k+1}^{HH,M}=\hat{p}_{k+1}^{HH,M}{(1-s_{H})}^{2}/W_{k+1}$

$p_{k+1}^{HB,M}=\hat{p}_{k+1}^{HB,M}(1-s_{H})(1-s_{B})/W_{k+1}$

$p_{k+1}^{BB,M}=\hat{p}_{k+1}^{BB,M}{(1-s_{B})}^{2}/W_{k+1}$

$(p_{k+1}^{HR,F},p_{k+1}^{HR,M})=(\hat{p}_{k+1}^{HR,F},\hat{p}_{k+1}^{HR,M})(1-s_{H})(1-s_{R})/W_{k+1}$

$(p_{k+1}^{HW,F},p_{k+1}^{HW,M})=(\hat{p}_{k+1}^{HW,F},\hat{p}_{k+1}^{HW,M})(1-s_{H})/W_{k+1}$

$(p_{k+1}^{RR,F},p_{k+1}^{RR,M})=(\hat{p}_{k+1}^{RR,F},\hat{p}_{k+1}^{RR,M}){(1-s_{R})}^{2}/W_{k+1}$

$(p_{k+1}^{RB,F},p_{k+1}^{RB,M})=(\hat{p}_{k+1}^{RB,F},\hat{p}_{k+1}^{RB,M})(1-s_{R})(1-s_{B})/W_{k+1}$

$(p_{k+1}^{RW,F},p_{k+1}^{RW,M})=(\hat{p}_{k+1}^{RW,F},\hat{p}_{k+1}^{RW,M})(1-s_{R})/W_{k+1}$

$(p_{k+1}^{BW,F},p_{k+1}^{BW,M})=(\hat{p}_{k+1}^{BW,F},\hat{p}_{k+1}^{BW,M})(1-s_{B})/W_{k+1}$

${(p}_{k+1}^{WW,F}{,p}_{k+1}^{WW,M})=(\hat{p}_{k+1}^{WW,F},\hat{p}_{k+1}^{WW,M})/W_{k+1}$

Here, $W_{k+1}$ is a normalizing term given by,

$$W_{k+1}=\left( \hat{p}_{k+1}^{HH,F}+\hat{p}_{k+1}^{HB,F}+\hat{p}_{k+1}^{BB,F} \right)\left( 1-s_{HH,F} \right)+\hat{p}_{k}^{HH,M}\left( 1-s_{H} \right)^{2}+\hat{p}_{k+1}^{HB,M}\left( 1-s_{H} \right)\left( 1-s_{B} \right)+\hat{p}_{k+1}^{BB,M}{(1-s_{B})}^{2}+\left( \hat{p}_{k+1}^{HR,F}+\hat{p}_{k+1}^{HR,M} \right)\left( 1-s_{H} \right)\left( 1-s_{R} \right)+\left( \hat{p}_{k+1}^{HW,F}+\hat{p}_{k+1}^{HW,M} \right)\left( 1-s_{H} \right)+\left( \hat{p}_{k+1}^{RR,F},\hat{p}_{k+1}^{RR,M} \right)\left( 1-s_{R} \right)^{2}+\left( \hat{p}_{k+1}^{RB,F}+\hat{p}_{k+1}^{RB,M} \right)\left( 1-s_{R} \right)\left( 1-s_{B} \right)+\left( \hat{p}_{k+1}^{RW,F}+\hat{p}_{k+1}^{RW,M} \right)\left( 1-s_{R} \right)+\left( \hat{p}_{k+1}^{BW,F}+\hat{p}_{k+1}^{BW,M} \right)\left( 1-s_{B} \right)+\hat{p}_{k+1}^{WW,F}+\hat{p}_{k+1}^{WW,M}$$

Note that here, fitness costs are relative to the WW female and male genotypes.

The likelihood of the population cage data was calculated by assuming a multinomial distribution of individuals having each sex and marker phenotype, and by using the model predictions to generate expected proportions for each set of parameter values. I.e., by calculating the log likelihood,

$$\log L(\theta)\propto\sum_{i=1}^{9} \sum_{k=1}^{n_{i}} A_{f,i,k}\log\left( p_{k}^{HW,F}\left( \theta\right)+p_{k}^{HR,F}\left( \theta\right) \right)+A_{m,i,k}\log\left( p_{k}^{HW,M}\left( \theta\right)+p_{k}^{HR,M}\left( \theta\right) \right)+B_{f,i,k}\log\left( p_{k}^{HH,F}\left( \theta\right)+p_{k}^{HB,F}\left( \theta\right) \right)+B_{m,i,k}\log\left( p_{k}^{HH,M}\left( \theta\right)+p_{k}^{HB,M}\left( \theta\right) \right)+C_{f,i,k}\log\left( p_{k}^{WW,F}\left( \theta\right)+p_{k}^{RW,F}\left( \theta\right)+p_{k}^{BW,F}\left( \theta\right)+p_{k}^{RR,F}\left( \theta\right)+p_{k}^{RB,F}\left( \theta\right) \right)+C_{m,i,k}\log\left( p_{k}^{WW,M}\left( \theta\right)+p_{k}^{RW,M}\left( \theta\right)+p_{k}^{BW,M}\left( \theta\right)+p_{k}^{RR,M}\left( \theta\right)+p_{k}^{RB,M}\left( \theta\right) \right)+D_{f,i,k}\log\left( p_{k}^{BB,F}\left( \theta\right) \right)+D_{m,i,k}\log\left( p_{k}^{BB,M}\left( \theta\right) \right)$$

Here, $A_{f,i,k}$ and $A_{m,i,k}$ are the number of DsRed+, kh+ females and males (genotypes HW and HR) at generation $k$ in experiment $i$, respectively, $B_{f,i,k}$ and $B_{m,i,k}$ are the corresponding number of DsRed+, kh- females and males (genotypes HH and HB), respectively, $C_{f,i,k}$ and $C_{m,i,k}$ are the corresponding number of DsRed-, kh+ females and males (genotypes WW, RW, BW, RR and RB), respectively, and $D_{f,i,k}$ and $D_{m,i,k}$ are the corresponding number of DsRed-, kh- females and males (genotype BB), respectively. The $i$th experiment is run for $n_{i}$ generations, and the expected genotype frequencies are dependent on the model parameters, $\theta=\{c,p_{HDR},p_{RES},s_{H}, s_{R},s_{B},s_{HH,F},p_{MC},p_{MR}\}$, as defined earlier.

Given prior knowledge that the HH, HB and BB genotypes confer infertility in females, we assumed that $s_{HH,F}=1$. All fitness costs were manifest after phenotype scoring, i.e. in the contribution of gametes to the next generation. The initial condition for the three 1:1 experiments was 50 HW males, 50 WW males, and 100 WW females (i.e. the drive system was introduced only in males). For the three 0.33:1 experiments, the initial condition was 25 HW males, 75 WW males, and 100 WW females, and for the three 0.1:1 experiments, the initial condition was 9 HW males, 90 WW males, and 100 WW females.

The value of $p_{HDR}$ was allowed to vary depending on whether the HW individual was female or male ($p_{HDR,F}$ in females, and $p_{HDR,M}$ in males), as suggested by the G0 cross data. As identifiability was an issue with estimating parameter values, we fixed the values of $c$, $p_{HDR,F}$ and $p_{HDR,M}$, based on G0 crosses that provided direct information on them, and fixed the value of $s_{HH,F}$ as mentioned previously. We used a Markov chain Monte Carlo (MCMC) sampling procedure to estimate the remaining parameters, $p_{RES}$, $s_{H}$, $s_{R}$, $s_{B}$, $p_{MC}$ and $p_{MR}$, including 95% credible intervals for them. The resulting model fits are depicted in Figs. Smod S2-S5.

**Stochastic Model Implementation.**

A stochastic version of the fitted model was implemented using a discrete generation version of the Mosquito Gene Drive Explorer model (MGDrivE) [36]) with an adult population size of 600. At each generation, for a given adult population, adult females mate with males, thereby obtaining a composite mated genotype (their own, and that of their mate) with mate choice following a multinomial distribution determined by adult male genotype frequencies multiplied by their lifetime relative mating competitiveness (informed by model fitting). Egg production by mated adult females then follows a Poisson distribution, proportional to the genotype-specific lifetime fecundity of the adult female (informed by model fitting). Offspring genotype follows a multinomial distribution informed by the composite mated female genotype and the inheritance pattern of the gene drive system. The inheritance pattern is captured by the “inheritance cube” module of MGDrivE for an autosomal CRISPR-based homing system with two varieties of resistant allele and maternal deposition of Cas, and parameterized based on the results of the model fitting described above. Progeny are then sampled for marker phenotype scoring and to seed the next generation, with sampling based on a multivariate hypergeometric distribution. 100 simulations were run for each initial condition, with the resulting marker phenotype time-series recorded and compared to those observed. From this, the proportion of simulations for which the AsMCRkh2 system spreads to fixation leading to population collapse was recorded and compared to that observed.
