# Supplementary figures and images for: Experimental population modification of the malaria vector mosquito, Anopheles stephensi
Source: PLoS Genet. 2019 Dec 19;15(12):e1008440. doi: 10.1371/journal.pgen.1008440 (PMC6922335; doi:10.1371/journal.pgen.1008440)

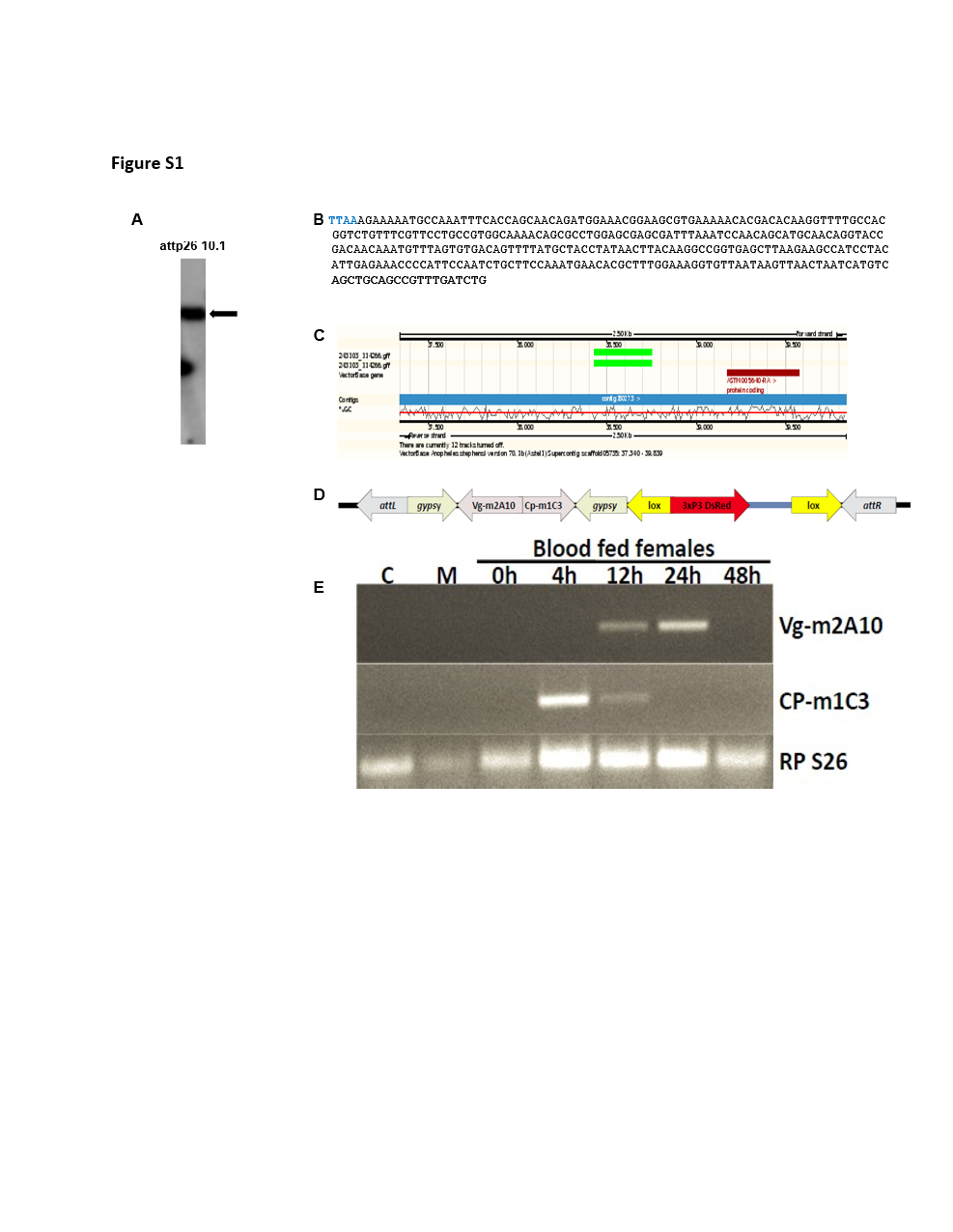

Supplement: S1 Fig — Characterization of the attp26 10.1 φC31 docking site line and AP26 dual effector line. A docking site line, attp26 10.1, carrying a φC31 attP nucleotide sequence for site‐specific integration, a gene encoding the cyan fluorescent protein under control of a 3xP3 gene promoter and enhancers, was generated using piggyBac‐mediated transformation and characterized following previously‐published protocols [31,32]. (A) Southern blot analysis of Bam HI‐digested attp26 10.1 genomic DNA probed with a 32P‐labelled gene amplification product derived from the cyan fluorescent protein (CFP) open reading frame. A single hybridizing fragment (arrow) shows that this is a single‐copy insertion into the An. stephensi genome. (B) Inverse polymerase chain reaction (IPCR) protocols were used to amplify a portion of the genomic DNA flanking the chromosomal insertion site of the attP docking site. The bolded TTAA represents the recognition site for piggy-Bac‐mediated transposition. (C) The nucleotide sequence of the IPCR amplicon was used to identify single scaffolds containing the sequence in each of the two versions of published An. stephensi genomes (scaffold 00077 from the Indian strain [60] and scaffold KB664547 from the SDA 500 strain [https://www.vectorbase.org]). (D) Schematic representation of the integrated transgenes in the dual effector line, AP26. The two single‐chain antibody constructs, Vg‐m2A10 and Cp‐m1C3, have been described [31]. They have been cloned between two gypsy sequences [26] and are flanked to one side by sites for lox‐mediated gene excision and the 3xP3‐DsRed marker gene. Integration of the construct into the docking site generates attL and attR sites. E) Reverse transcriptase polymerase chain reaction (RT‐PCR) analysis of the expression of the Vg‐m2A10 and Cp‐m1C3 transgenes following a bloodmeal. Gene‐specific primers were used to amplify samples of RNA prepared from males (M), and females at 0, 4, 12, 24 and 48 hours (h) after a blood meal. C is a control w [file pgen.1008440.s025.tif]

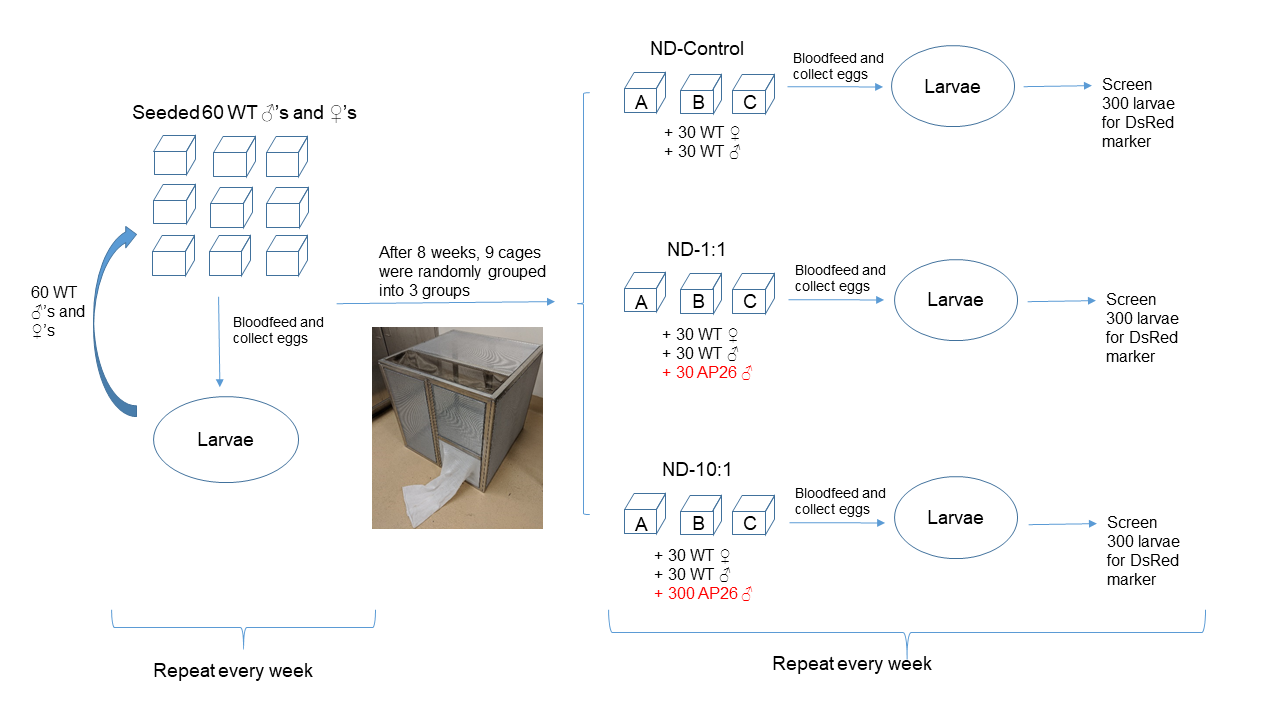

Supplement: S2 Fig — Sixty wild-type male and female larvae were added to each of nine 0.216 m3 cages (image). Beginning week 3, females were provided a bloodmeal weekly and eggs were collected and hatched. Sixty larvae were randomly selected and returned to their respective cages weekly until week 8 to create an age-structured population in the cages. Beginning week 9, the nine cages were randomly assigned in triplicate as ND-Control-A, B and C’, ‘ND-1:1-A, B and C’ and ‘ND-10:1-A, B and C’ AP26:wild-type male release ratios replicate trials. ‘ND’ refers to ‘non-drive’, 1:1 and 10:1 refer to transgenic to wild-type male release ratios, and ‘A’, ‘B’ and ‘C’ refer to the individual cage replicates. Females were again provided a bloodmeal weekly, and eggs were collected, hatched, and reared to pupae. 30 male and 30 female wild- type pupae were added back to their cages. Cages ND-Control-A, B and C had no additional pupae added. Cages ND 1:1 (A, B and C) had an additional 30 transgenic AP26 male pupae added. Cages ND-10:1-(A, B and C) had an additional 300 transgenic AP26 male pupae added over three days. 300 larvae from each of the nine cages were selected randomly and screened for the DsRed marker. This procedure was repeatedly weekly until week 22. (TIF) [file pgen.1008440.s026.tif]

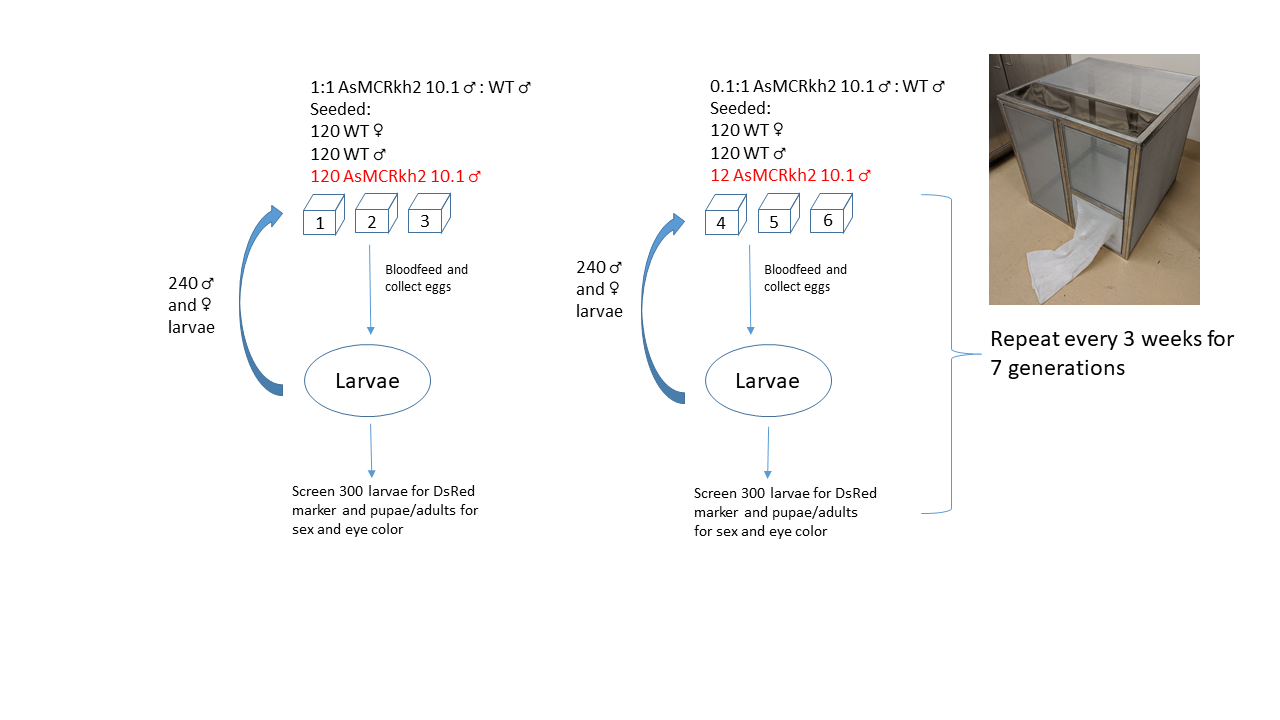

Supplement: S3 Fig — 120 wild-type males and 120 wild-type females were added to each of six 0.216 m3 cages (image). Cages OD-1:1 (A, B and C) with a 1:1 AsMCRkh2 male release ratio had an additional 120 transgenic AsMCRkh2 males added. Cages OD-0.1:1 (A, B and C) with a 0.1:1 male release ratio had an additional 12 transgenic AsMCRkh2 males added. Every 3 weeks for 7 generations, adult females were provided mice for bloodmeals and eggs were collected and hatched. 240 larvae were selected randomly and returned to their respective cages. No additional AsMCRkh2 males were added. 300 larvae were selected randomly and screened for the DsRed marker. They were later screened as pupae and adults for eye-color and sex. (TIF) [file pgen.1008440.s027.tif]

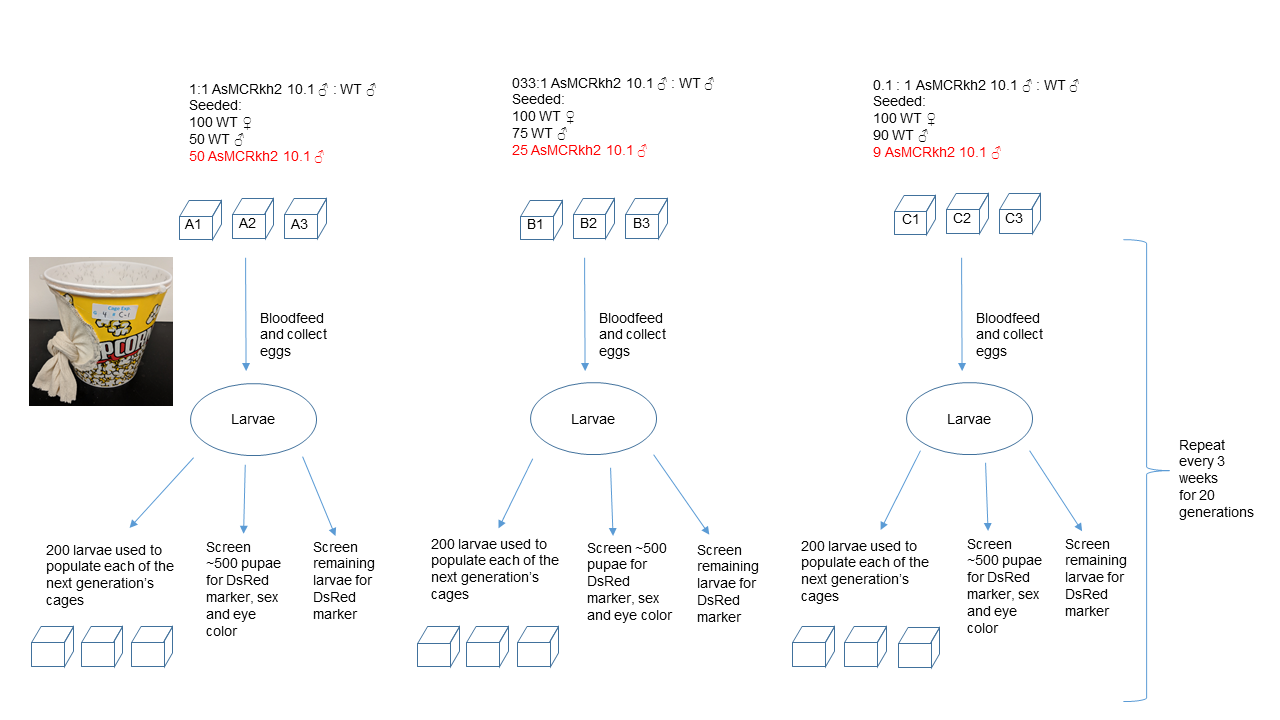

Supplement: S4 Fig — Nine small 0.005 m3 cages (image) were set up in triplicate according to their transgenic AsMCRkh2:wild-type male release ratios. Cages NOD-1:1 (A, B and C) with a 1:1 male release ratio had 100 wild-type females, 50 wild-type males, and 50 AsMCRkh2 males added. Cages NOD-0.33:1 (A, B and C) with a 0.33:1 male release ratio had 100 wild-type females, 75 wild-type males, and 25 AsMCRkh2 males added. Cages NOD-0.1:1 (A, B and C) with a 0.1:1 male release ratio had 100 wild-type females, 90 wild-type males, and 9 AsMCRkh2 males added. Females were provided a bloodmeal and eggs were collected and hatched. For Cages NOD-1:1 (A, B and C) and NOD-0.33:1 (A, B and C), 200 larvae were selected randomly and used to populate new cages, separate from that of their parents, for the next generation. An additional 500 larvae were selected randomly and reared to pupae, when they were screened for the DsRed marker and eye-color. The 500 pupae were then reared to adults and scored by sex. All remaining larvae were screened for the DsRed marker. This procedure was repeated every 3 weeks for 20 generations. For Cages NOD-0.1:1 (A, B and C) in generations 1–12, all larvae were scored for the DsRed marker and 200 larvae reflecting the existing transgene frequency were used to populate new cages. Beginning generation 13, Cages NOD-0.1:1 (A, B and C) were set up identically to Cages NOD-1:1 (A, B and C) and NOD-0.33:1 (A, B and C). (TIF) [file pgen.1008440.s028.tif]

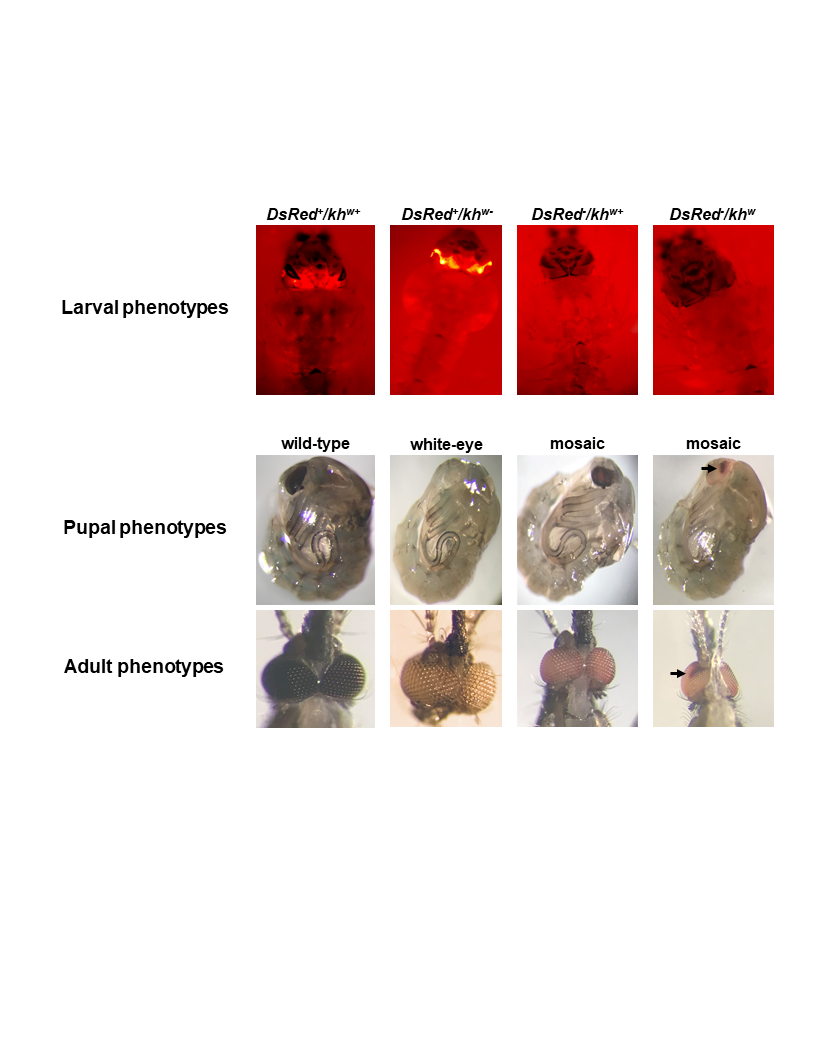

Supplement: S5 Fig — Fluorescent and bright-field images of a larva, pupa and adult. Larvae were screened for the DsRed phenotype (DsRed+ and DsRed-). Pupae and adults were screened for the eye color phenotypes (black eye kh+, white eye khw and mosaic khmosiac) and sex (♂ and ♀). The black arrow indicates a patch of colored-cells in the white background of the mosaic eye. (TIF) [file pgen.1008440.s029.tif]

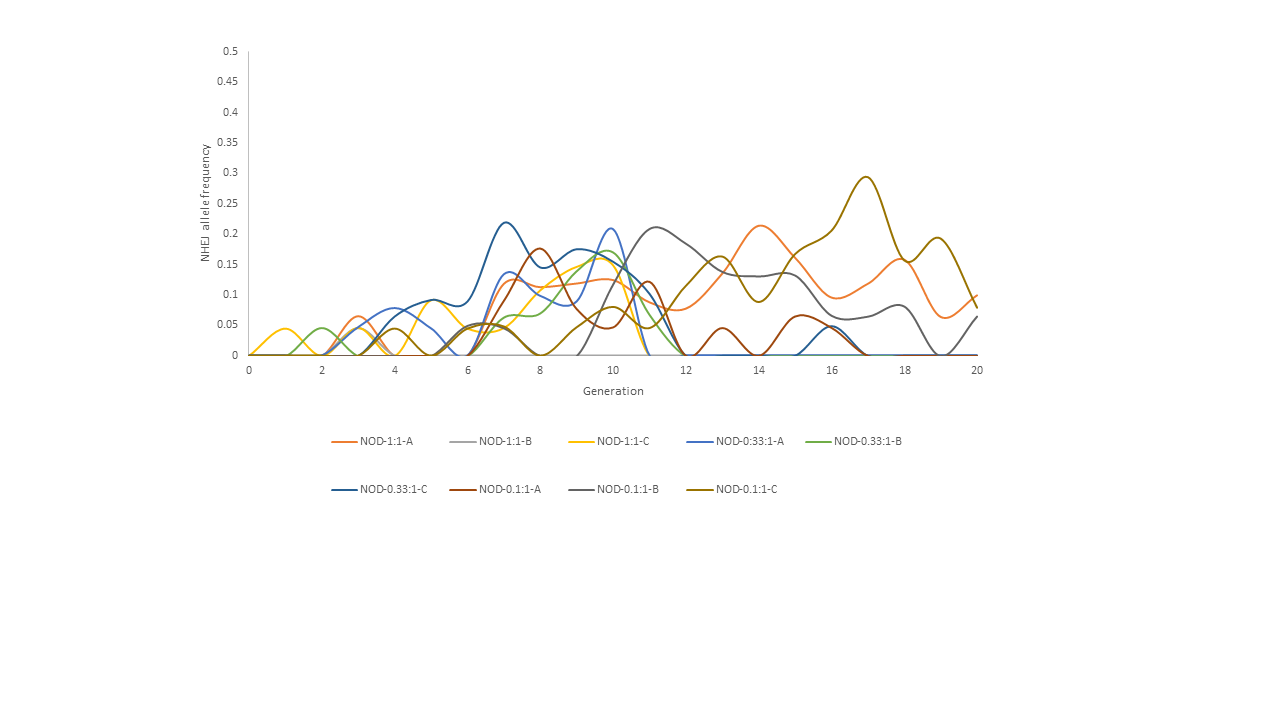

Supplement: S6 Fig — White-eyed/DsRed- phenotype mosquitoes contained two (same or different) NHEJ alleles that disrupted kynurenine hydroxylase enzymatic activity and resist endonuclease cutting and homing events. The allele frequency was calculated from the observed white-eyed/DsRed- individuals from ~500 mosquitoes screened for eye color phenotype. The initial NHEJ allele frequency was ~ 5% per generation in all cages. (TIF) [file pgen.1008440.s030.tif]

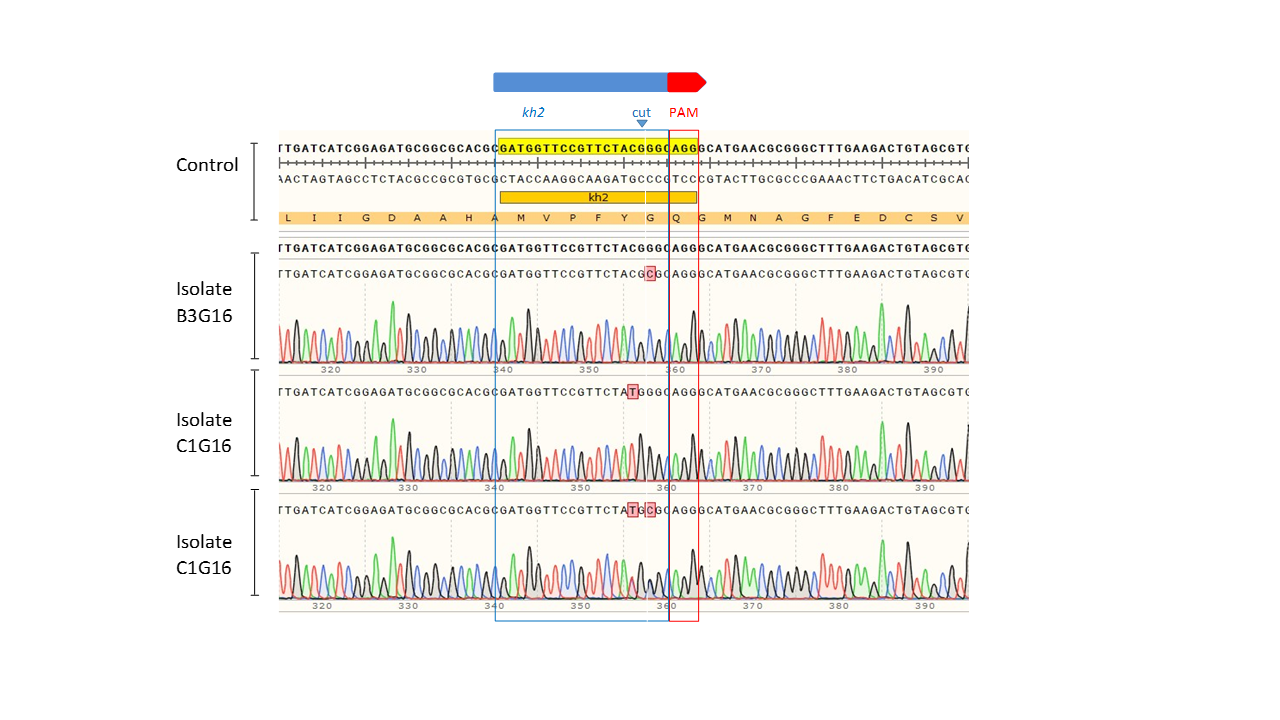

Supplement: S7 Fig — The top sequence is the wild-type reference sequence at the target kh2 site in control mosquitoes. The PAM sequence is in the red box, the gRNA targeted sequence is in the blue box, and the gRNA-directed cleavage site is indicated by a vertical thin white line. Sequencing randomly selected samples of black-eyed DsRed-negative mosquitoes from each cage revealed that there were two mutations that were homozygous in these mosquitoes. A point mutation of 1181C>T led to a silent mutation of Y328 while 1183G>C results in a substitution (G329A). Sequenced mosquitoes showed that some were homozygous for either mutation while others were heterozygous for both. These mutations conserved the kynurenine hydroxylase enzymatic activity while preventing endonuclease cutting. (TIF) [file pgen.1008440.s031.tif]

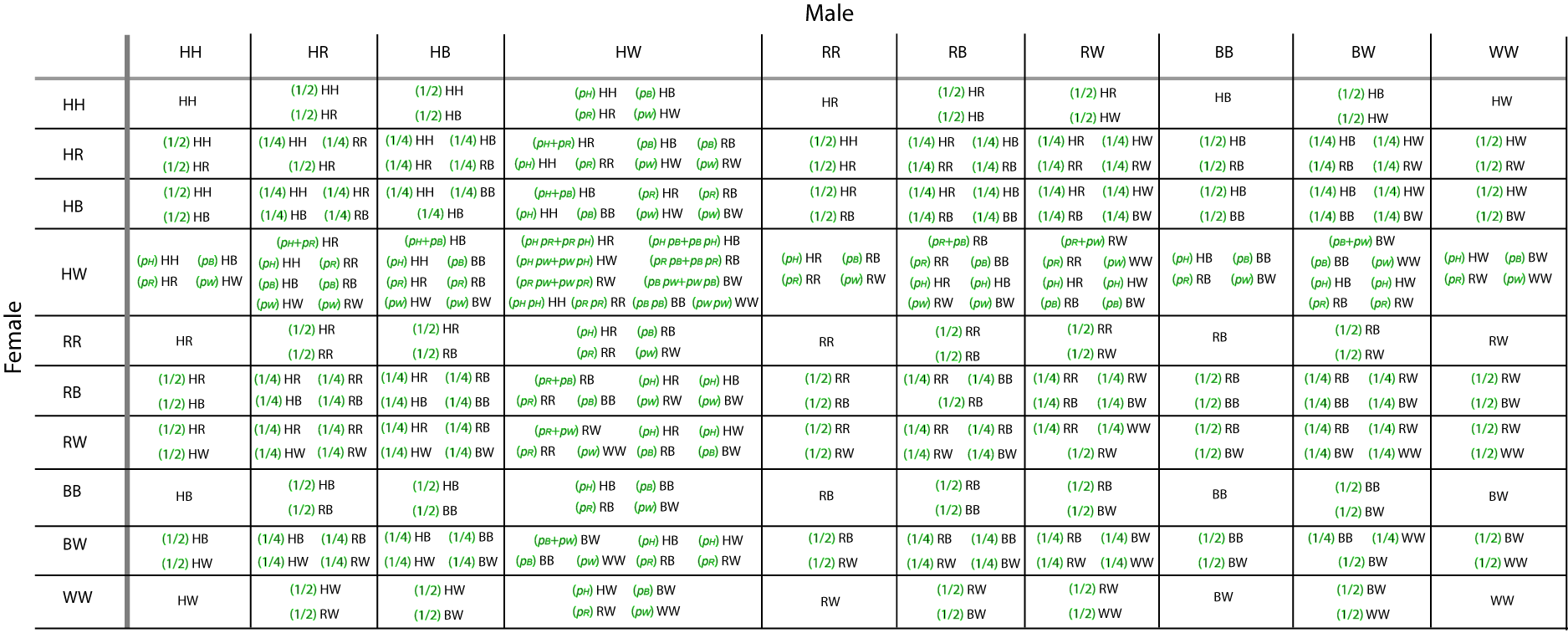

Supplement: S1 File — “H” denotes the autosomal AsMCRkh2 homing gene drive system, “W” denotes the wild-type allele targeted by the homing system, “R” denotes an in-frame, cost-free homing-resistant allele, and “B” denotes an out-of-frame or otherwise costly “broken” homing-resistant allele. Alleles segregate in a Mendelian fashion, with the exception of the W allele of HW heterozygotes. A proportion, pH = 0.5(1 + cpHDR), of the gametes produced by HW heterozygotes are H alleles, where half are already H alleles, and a proportion, c, of the W alleles are cleaved, with a proportion, pHDR, of those being subject to accurate homology-directed repair (HDR) and becoming H alleles. The rate of HDR is sex-specific–i.e. there is a value, pHDR,F, in females, and a value, pHDR,F, in males. Of the cleaved W alleles that do not become H alleles, a proportion, pRES, become R alleles, while the remainder, 1 − pRES, become B alleles. I.e., a proportion, pR = 0.5c(1 − pHDR)pRES, of the W alleles of HW heterozygotes become R alleles, while a proportion, pB = 0.5c(1 − pHDR)(1 − pRES), become B alleles. Finally, a proportion, 1 − c, of W alleles are not cleaved, and hence the proportion of W gametes produced by HW heterozygotes is pW = 0.5(1 − c). Subsequent to fertilization, and not depicted here, the effects of maternal deposition of Cas are accommodated. This can lead to the W allele of an offspring being cleaved if the mother has the H allele, allowing Cas to be deposited in the embryo. We consider cleavage to occur in a proportion, pMC, of these embryos, with a proportion, pMR, of the cleaved W alleles become R alleles, and the remainder, 1 − pMR, becoming B alleles. This, and the inheritance pattern depicted here, are described fully in the supplementary S1 Text. (TIF) [file pgen.1008440.s032.tif]

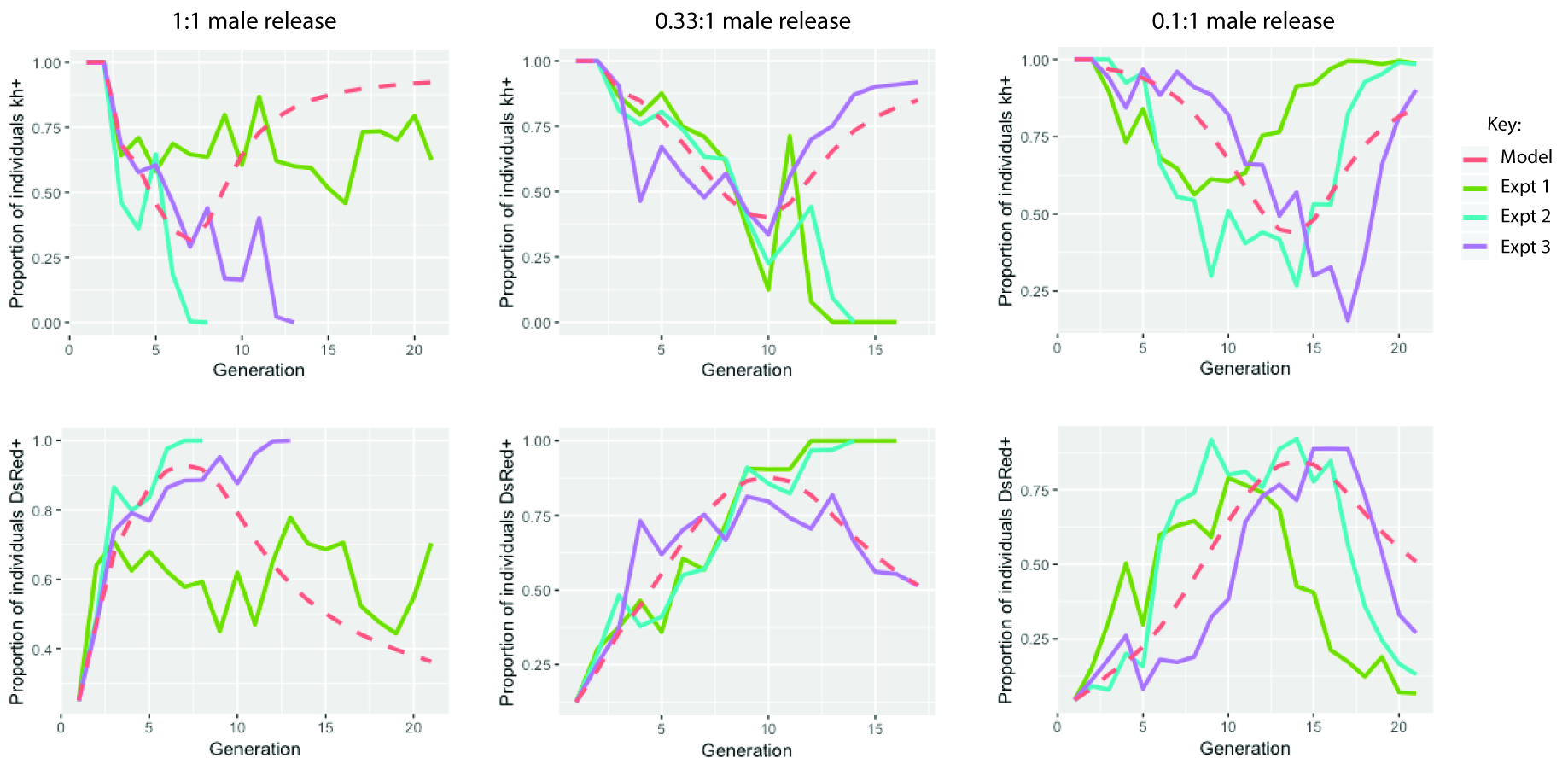

Supplement: S2 File — Observed and predicted dynamics with respect to the DsRed and kh marker phenotypes for non-overlapping generation experiments with the AsMCRkh2 gene drive system. Experiments were set up with 100 wild-type (WW, where W represents the wild-type allele) females, and 100 or 99 males. For 1:1 releases (left), the initial condition is 50 transgenic males heterozygous for the drive system (HW, where H represents the homing-based drive system) and 50 WW males, for 0.33:1 releases (middle), the initial condition is 25 HW males and 75 WW males, and for 0.1:1 releases (right), the initial condition is 9 HW males and 90 WW males. Population counts were monitored over 21 generations for the 1:1 and 0.1:1 releases, and over 17 generations for the 0.33:1 releases. Results from these experiments are shown as solid lines (3 experiments per release ratio), with fitted model predictions shown as dashed lines (1 simulation per release ratio). Observed data are consistent with homing efficiencies inferred from generation G0, namely an accurate homing efficiency of 95% in females and 98% in males, and with 0.5% (95% CrI: 0.0–3.6%) of resistant alleles being in-frame, cost-free (R), and the remainder being out-of-frame or otherwise costly resistant “broken” alleles (B). Furthermore, maternal deposition of Cas is inferred to result in cleavage of embryonic W alleles with a frequency of 70% (95% CrI: 68–72%), with 22% (95% CrI: 21–24%) of the cleaved W alleles becoming R alleles, and the remainder becoming B alleles. Given these rates, the data are consistent with the following fitness costs: females having two copies of the homing and/or broken resistant allele (HH, HB or BB) are infertile, otherwise the H, R and B alleles have multiplicative fitness costs per copy of 7.9% (95% CrI: 7.4–8.6%), 18.4% (95% CrI: 17.7–19.1%), and 0.0% (95% CrI: 0.0–0.0%), respectively. The DsRed+ phenotype is associated with having at least one copy of the gene drive allele (i.e. genotypes HH, HR, HB and HW), [file pgen.1008440.s033.tif]

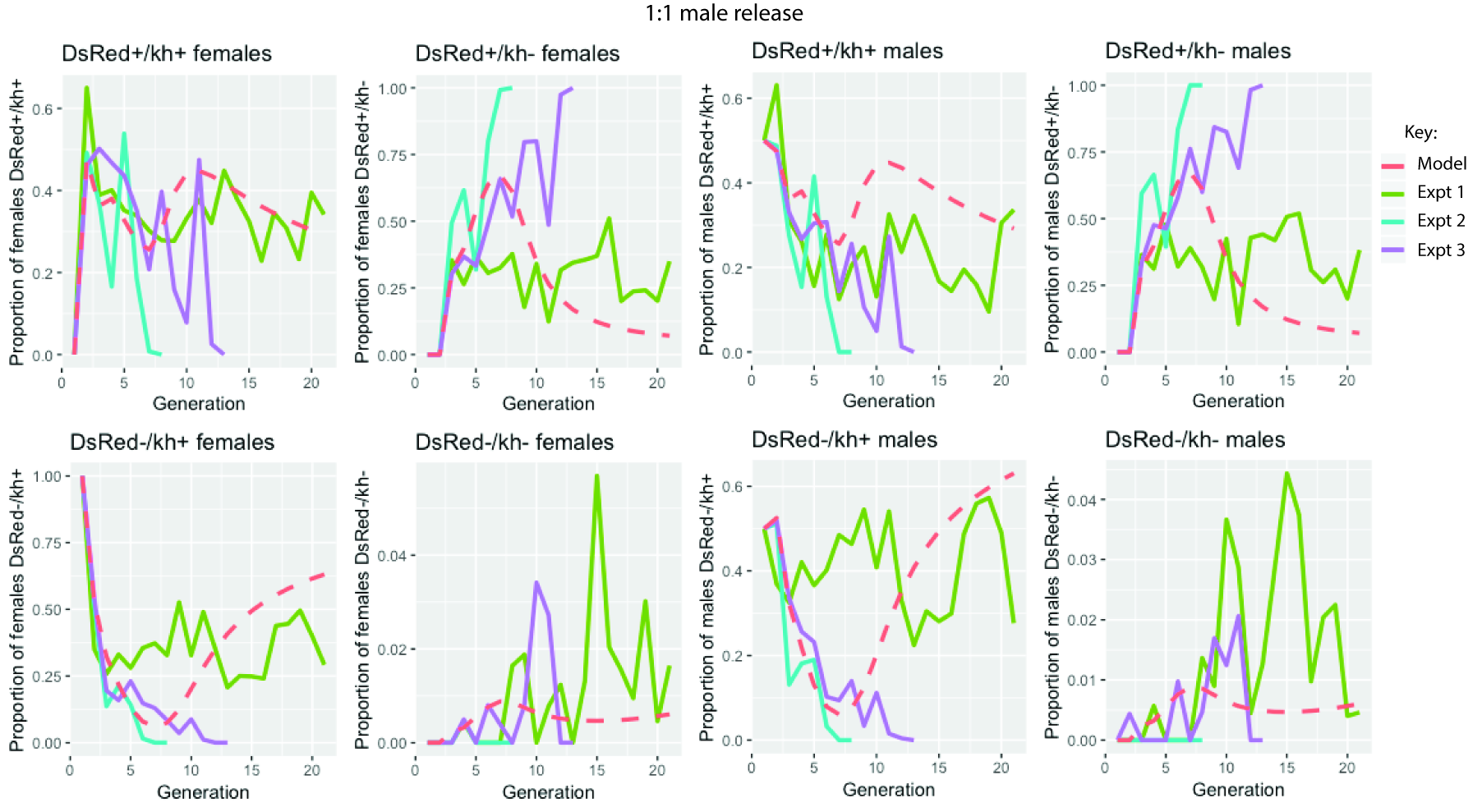

Supplement: S3 File — Observed and predicted dynamics with respect to DsRed and kh marker phenotype combinations for 1:1 non-overlapping generation experiments with the AsMCRkh2 gene drive system. Experiments were set up with 100 wild-type (WW, where W represents the wild-type allele) females, 50 transgenic males heterozygous for the drive system (HW, where H represents the homing-based drive system) and 50 WW males. Population counts were monitored over 21 generations for experiment 1, although the population crashed earlier than this for experiments 2 and 3. Results from these experiments are shown as solid lines, with fitted model predictions as dashed lines. Model predictions are based on data from all 9 experiments (3 for each release ratio: 1:1, 0.33:1 and 0.1:1 HW:WW males), with estimated and inferred parameter values described in the Results section of the manuscript. DsRed+/kh- individuals have the gene drive (H) allele, but not the wild-type (W) or in-frame resistant (R) allele (i.e. genotypes HH and HB). These genotypes spread to fixation in experiments 2 and 3, but stagnate in experiment 1. DsRed-/kh- individuals lack both the H allele and the W or R allele (i.e. genotype BB). This genotype persists at low levels due to being consistently generated, but conferring infertility in females. DsRed+/kh+ individuals have both the H allele and the W or R allele (i.e. genotypes HW and HR), and DsRed-/kh+ individuals have the W or R allele, but lack the H allele (i.e. genotypes WW, RW, BW, RR and RB). In both cases, these genotypes persist in experiment 1; but are eliminated in experiments 2 and 3 as the H and/or B alleles spread to fixation. (TIF) [file pgen.1008440.s034.tif]

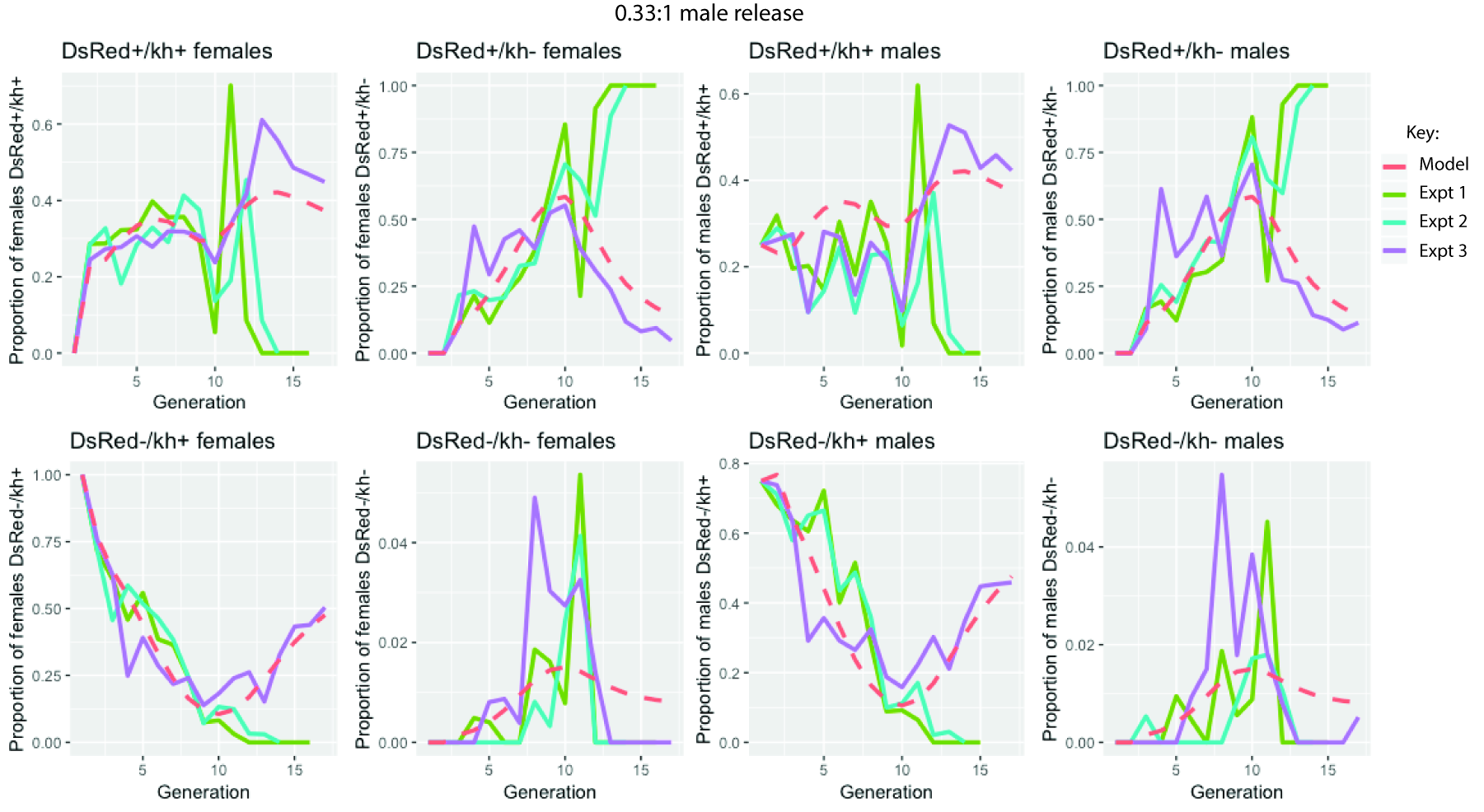

Supplement: S4 File — Observed and predicted dynamics with respect to DsRed and kh marker phenotype combinations for 0.33:1 non-overlapping generation experiments with the AsMCRkh2 gene drive system. Experiments were set up with 100 wild-type (WW, where W represents the wild-type allele) females, 25 transgenic males heterozygous for the drive system (HW, where H represents the homing-based drive system) and 75 WW males. Population counts were monitored over 17 generations. Results from these experiments are shown as solid lines, with fitted model predictions as dashed lines. Model predictions are based on data from all 9 experiments (3 for each release ratio: 1:1, 0.33:1 and 0.1:1 HW:WW males), with estimated and inferred parameter values described in the Results section of the manuscript. DsRed+/kh- individuals have the gene drive (H) allele, but not the wild-type (W) or in-frame resistant (R) allele (i.e. genotypes HH and HB). These genotypes spread to fixation in experiments 1 and 2, but stagnate in experiment 3. DsRed-/kh- individuals lack both the H allele and the W or R allele (i.e. genotype BB). This genotype persists at low levels due to being consistently generated, but conferring infertility in females. DsRed+/kh+ individuals have both the H allele and the W or R allele (i.e. genotypes HW and HR), and DsRed-/kh+ individuals have the W or R allele, but lack the H allele (i.e. genotypes WW, RW, BW, RR and RB). These genotypes persist in experiment 3; but are eliminated in experiments 1 and 2 as the H and/or B alleles spread to fixation. (TIF) [file pgen.1008440.s035.tif]

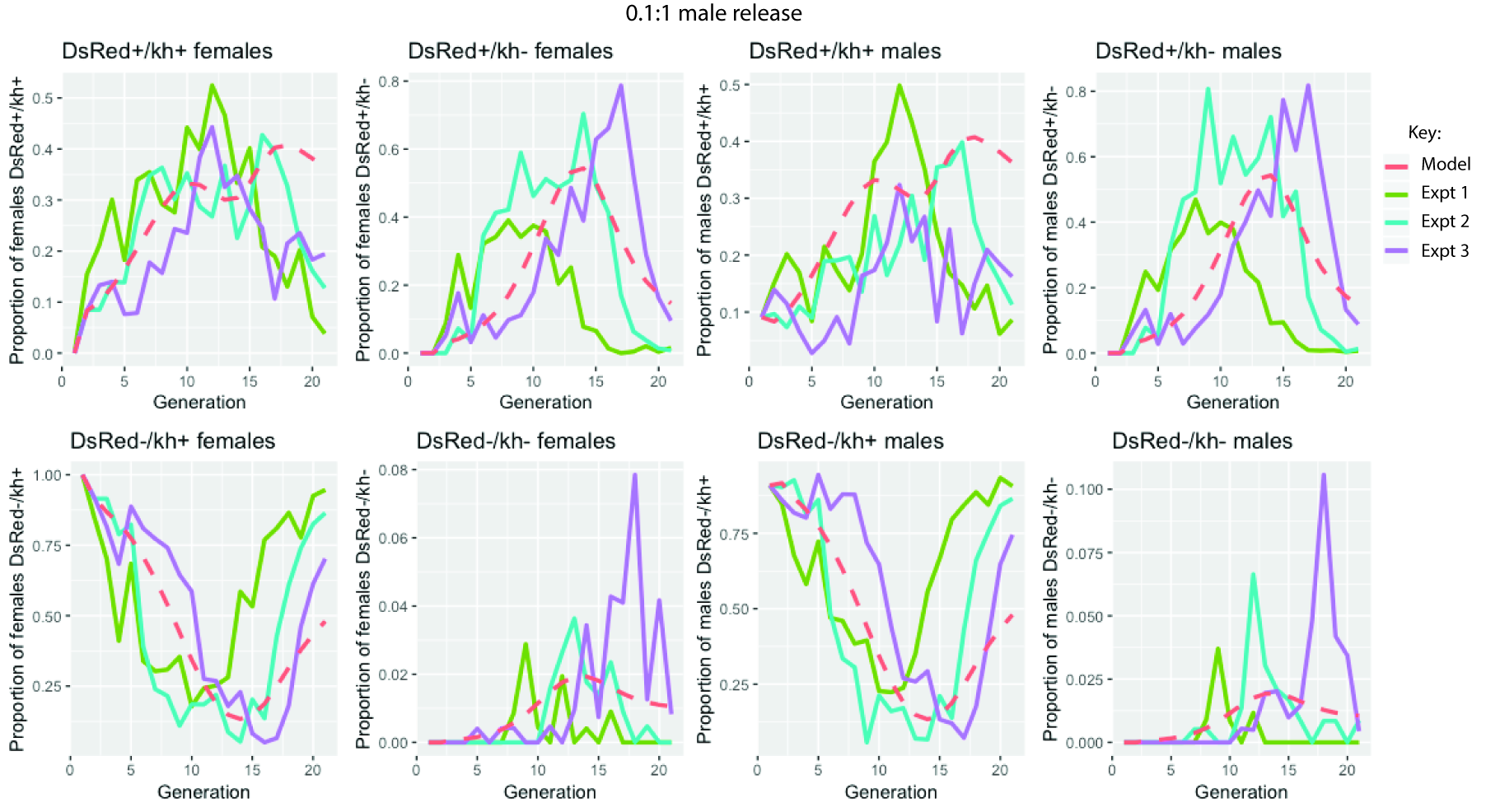

Supplement: S5 File — Observed and predicted dynamics with respect to DsRed and kh marker phenotype combinations for 0.1:1 non-overlapping generation experiments with the AsMCRkh2 gene drive system. Experiments were set up with 100 wild-type (WW, where W represents the wild-type allele) females, 9 transgenic males heterozygous for the drive system (HW, where H represents the homing-based drive system) and 90 WW males. Population counts were monitored over 21 generations. Results from these experiments are shown as solid lines, with fitted model predictions as dashed lines. Model predictions are based on data from all 9 experiments (3 for each release ratio: 1:1, 0.33:1 and 0.1:1 HW:WW males), with estimated and inferred parameter values described in the Results section of the manuscript. DsRed+/kh- individuals have the gene drive (H) allele, but not the wild-type (W) or in-frame resistant (R) allele (i.e. genotypes HH and HB). These genotypes spread to ~40–80% in the experiments, and to ~55% in the simulation, before declining. DsRed-/kh- individuals lack both the H allele and the W or R allele (i.e. genotype BB). This genotype persists at low levels due to being consistently generated, but conferring infertility in females. DsRed-/kh+ individuals have the W or R allele, but lack the H allele (i.e. genotypes WW, RW, BW, RR and RB). These genotypes decline in frequency initially, and then begin to rise again beginning in generations 12–17, suggesting a resurgence of the R and/or W allele. Perhaps as a consequence of this, DsRed+/kh+ individuals having both the H allele and the W or R allele (i.e. genotypes HW and HR) persist for the duration of the experiments. (TIF) [file pgen.1008440.s036.tif]
